# Mapping the prevalence of soil-transmitted helminth infections in the Western Pacific Region: a spatial modelling study

**DOI:** 10.1016/j.lanwpc.2025.101581

**Published:** 2025-05-28

**Authors:** Beth Gilmour, Haileab Fekadu Wolde, Kinley Wangdi, Angela Cadavid Restrepo, Tsheten Tsheten, Matthew Kelly, Archie C.A. Clements, Darren Gray, Colleen L. Lau, Fe Esperanza Espino, Susana Vaz Nery, Adam W. Bartlett, Alemneh Mekuriaw Liyew, Temesgen Yihunie Akalu, Kefyalew Addis Alene

**Affiliations:** a School of Population Health, Faculty of Health Sciences, Curtin University, WA, Australia; b Geospatial and Tuberculosis Research Team, The Kids Research Institute Australia, WA, Australia; c Institute of Public Health, College of Medicine and Health Sciences, University of Gondar, Gondar, Ethiopia; d HEAL Global Research Centre, Health Research Institute, Faculty of Health, University of Canberra, Canberra, ACT, Australia; e National Centre for Epidemiology and Population Health, Australian National University, Canberra, ACT, Australia; f School of Public Health, The University of Queensland, Brisbane, QLD, Australia; g Queens University Belfast, Belfast, United Kingdom; h QIMR Berghofer Medical Research Institute, Brisbane, QLD, Australia; i Centre for Clinical Research, The University of Queensland, Brisbane, QLD, Australia; j Research Institute for Tropical Medicine, Department of Health, Philippines; k The Kirby Institute, University of New South Wales, NSW, Australia

**Keywords:** Soil-transmitted helminth, STH, Western Pacific, Spatial, Prediction, Mapping

## Abstract

**Background:**

Soil-Transmitted Helminth (STH) infections are a significant health issue in the Western Pacific Region (WPR). This study aims to produce high-resolution spatial prediction STH prevalence maps for the WPR.

**Methods:**

Bayesian model-based geostatistical frameworks were developed for each STH species (*Ascaris lumbricoides*, *Trichuris trichiura*, *Strongyloides stercoralis*, and hookworm) to estimate infection prevalence at a spatial resolution of 1 km^2^. A systematic review created a comprehensive database of STH prevalence surveys, which informed the geostatistical frameworks. Logistic regression models incorporating both fixed covariate effects and spatial random effects were applied to identify drivers of spatial distribution for each species.

**Findings:**

We analysed 227 surveys from 3122 locations across 15 countries in the WPR. Between 1998–2011 and 2012–2021 substantial reductions in the pooled prevalence of hookworm (21.3%–3.7%), *A. lumbricoides* (21.7%–6.5%) and *T. trichiura* (22.5%–9.7%) were observed, while *S. stercoralis* prevalence increased (13.3%–18.4%). High-resolution spatial prediction maps revealed notable geographical variations in STH prevalence, with persistent hotspots identified in China, Cambodia, Malaysia, and Vietnam. Altitude and distance to health facilities were positively associated with the prevalence of hookworm and *A. lumbricoides*, while sand content in soil was positively associated with all STH species. In contrast, coarse soil fragments and organic carbon content were negatively associated with the prevalence of *T. trichiura* and *A. lumbricoides*.

**Interpretation:**

The high-resolution spatial prediction maps produced in this study can inform resource prioritization to accelerate STH elimination efforts.

**Funding:**

10.13039/501100000925National Health and Medical Research Council (1153727 ACE-NTD).

**Research in context**

**Evidence before this study**

Soil-transmitted helminth (STH) infections, which cause significant suffering and disability, are estimated to impact 1.5 billion people globally. These infections which have their greatest impact on disadvantaged and vulnerable populations, are classified as a Neglected Tropical Disease (NTD). The World Health Organization (WHO) 2021–2030 NTD Roadmap and the United Nations (UN) Sustainable Development Agenda are aligned in their targets to eliminate NTD infections. To achieve the 2030 targets, understanding NTD epidemiology and the burden of these infections have been identified as a priority and the WHO have recommended new mapping tools be developed to identify areas of risk. Although previous studies have mapped STH infections, there is a lack of information at a high level of spatial resolution which is key to optimizing strategies and resources to achieve the 2030 targets.

**Added value of this study**

This study produces the first high-resolution spatial prediction prevalence maps for *Ascaris lumbricoides, Trichuris trichiura, Strongyloides stercoralis*, and hookworm infection across the Western Pacific Region (WPR). For each STH species, the study integrated environmental and socioeconomic covariates into Bayesian geostatistical frameworks to identify key drivers of infection distribution. The results showed substantial spatial variation in infection prevalence and facilitated infection risk prediction in areas lacking prevalence data. The study identified a trend of increased *Strongyloides* prevalence over time and found indigenous and ethnic minorities to carry a disproportionately high level of STH infection, which calls for the prioritization of this population group in control programmes. This modelling study builds on geospatial analyses techniques and delivers results that can contribute to STH elimination.

**Implications of all the available evidence**

The results suggest significant progress has been made in reducing the prevalence of hookworm, *A. lumbricoides*, and *T. trichiura* through control programs in the WPR, while the persistent hotspots in China, Cambodia, Malaysia, and Vietnam highlight the need for geographically targeted strategies to accelerate the elimination of STH as a public health problem. The results provide important information on high-risk areas for STH infection in the WPR and can be used to prioritize the identification of vulnerable populations in the region. To maximize the utility of these analytics, ongoing survey data collection is required. Geospatial analysis and prevalence surveys can work hand-in-hand to inform each other and augment their respective contributions.

## Introduction

Soil-transmitted helminth (STH) infections are estimated to impact 1.5 billion people,^1^ a figure that equates to 19% of the world's population. Soil-transmitted helminthiases are classified as Neglected Tropical Diseases (NTDs), due to the significant suffering and disability they impose, despite the fact that they can be controlled and their impact mitigated.^2^ In 2020, the magnitude of health loss due to NTDs was estimated at 26 million disability-adjusted life years (DALYs), with STHs accounting for the greatest burden at 5.2 million DALYs.^3^

The four species of STH most commonly referred to include *Ascaris lumbricoides* (roundworms), *Trichuris trichiura* (whipworms), *Necator americanus* and *Ancylostoma duodenale* and the zoonotic *Ancylostoma ceylanicum* (hookworms).^4^ These parasites prevail in low- and middle-income countries located within the tropics and subtropics.^1^^,^^5^ Another STH of significance is *Strongyloides stercoralis,* but its prevalence can be hard to quantify because traditional diagnostic methods lack sensitivity and patients are often asymptomatic.^6^ *S. stercoralis* prevails in both tropical and temperate climates,^7^ and is differentiated from the other STH species by its auto-infective lifecycle.^8^

STHs have their greatest impact on disadvantaged populations where hygiene and sanitation are inadequate.^1^ Infection can result in chronic and debilitating morbidity, the extent of which is directly related to worm burden.^9^ Symptoms of STH infection are often hard to diagnose due to the effects of poverty, malnutrition, and concurrent disease, which are often common among those worst affected by infection.^10^ Despite these confounding factors, a number of morbidities have been well documented including malnutrition, anaemia, vitamin A deficiency, impaired growth and physical development, intestinal obstruction, and poor cognitive and intellectual development.^1^^,^^11^

The World Health Organization (WHO) 2021–2030 NTD Roadmap sets global targets to reduce the burden of 20 prioritized NTDs which include STHs.^12^ The roadmap is aligned with target 3.3 in the United Nations (UN) Sustainable Development Agenda which seeks to end a number of epidemics, including NTDs, by 2030.^12^^,^^13^ Although significant progress was made in the initial WHO roadmap 2012–2020,^12^ progress towards 2030 targets is off-track and challenged by persistent underlying risk factors e.g., poverty, population growth and climate change.^13^

Key to the WHO STH control strategy is targeted preventative chemotherapy (PC) to at risk populations twice a year in areas where STH prevalence is ≥50% and once a year where prevalence is 20–49%.^14^^,^^15^ Populations considered at risk include pre-school aged children (pre-SAC), school-aged children (SAC) and women of reproductive age,^1^ who are at increased risk due to their susceptibility to morbidity. Workers engaged in certain occupations are also considered at risk due to increased environmental infection exposure.^1^ Successful STH elimination will however require health and hygiene education and the provision of adequate sanitation, with modelling and experimental studies suggesting the need to also extend PC to the whole community.^1^^,^^16^, ^17^, ^18^

Understanding NTD epidemiology and the burden of infection has been identified as one of the actions required to achieve the 2030 targets.^12^ In 2021, it is estimated that 1.6 billion people required interventions against NTDs globally, with 72 million of these residing within the WHO Western Pacific Region (WPR).^19^ In 2020, it was estimated that 43% of the 1022 million people requiring PC to treat STH globally, received treatment.^20^ It is approximated that 171 million pre-SAC and SAC required PC in the WPR in 2021 and that 32% of these received treatment.^21^

Understanding the distribution of infection prevalence is key to maximizing control efforts, but in countries where NTDs prevail, prevalence surveys are often limited and/or are not feasible due to resource constraints. Spatial predictive mapping provides disease prevalence estimates at a small scale of resolution thereby identifying “hotspots”, in addition to being able to provide prevalence estimates where empiric parasitological data are limited. This analytical approach, which combines systematic review, meta-analysis, and geospatial analysis principles, delivers results that can be used to prioritize disease control efforts. Prediction mapping has been successfully applied to a number of diseases including HIV,^22^ TB,^23^ malaria,^24^, ^25^, ^26^ cholera,^27^ and dengue,^28^ and has been applied to STHs in South America and South Africa.^29^, ^30^, ^31^ This study utilizes the same analytical approach to evaluate the prevalence of STH infections within the WPR.

## Methods

Our methodological approach for estimating STH prevalence is largely consistent with previous studies.^29^^,^^32^ Full details are provided in the supplementary and a published protocol.^33^ In brief, our study integrated systematic review, meta-analysis, and geospatial analysis into a single framework to estimate the prevalence of infection for different STH species on a 1 km^2^ grid across countries in the WPR with available data. To pool infection prevalence at a country level, random effects meta-analysis was used, and countries were included in the analysis based on the availability of subnational STH prevalence survey data. The methods in this study focus on spatial prediction, as interpreting covariate effects in the presence of spatial random effects can be problematic due to potential spatial confounding.^34^

### Data sources

Data for this study were sourced from STH prevalence surveys published in peer-reviewed journals between January 2000 and September 2023. A systematic search, following PRISMA guidelines,^35^ was conducted across databases including PubMed, Scopus, ProQuest, Embase, and Web of Science, with additional searches in grey literature and regional databases. The WHO regional classification system was used to define the countries within the WPR,^36^ and the search used relevant MeSH terms and keywords for STH infections.

Two independent reviewers screened the studies, with eligibility criteria including the requirement for studies to be undertaken on human STH infections and conducted using random sampling in the WPR. For studies with both pre- and post-intervention surveys, only baseline pre-intervention data were extracted. Data extraction involved collecting details on study characteristics, participant demographics, and STH outcomes. The methodological quality of the included studies was evaluated using a modified version of the Newcastle–Ottawa Scale (NOS),^37^ with studies subsequently categorized as low, medium, or high quality. Details on the search strategy, study selection criteria, data collection tools, and quality assessment framework are provided in the Supplementary Table S1.

### Covariate data sources

Covariate data used for the geospatial model were obtained from publicly accessible sources. Data sources included WorldPop^38^ for population density data and the Malaria Atlas Project (MAP)^39^ for healthcare access data. Data on climatic variables such as precipitation, mean temperature, and solar radiation were obtained from the Global Climate Database,^40^ and data on altitude were derived from the Shuttle Radar Topography Mission (SRTM).^41^ Grided soil characteristics data were obtained from the International Soil Reference and Information Centre (ISRIC),^42^ and land cover data were extracted from the European space agency database.^43^ The database of Global Administrative Areas (GADM),^44^ was used to obtain polygon shapefiles for the administrative boundaries of each country. These covariates were chosen based on their established association with STH infections from prior studies,^32^^,^^45^^,^^46^ as well as the availability of high-resolution spatial data for countries in WPR. Supplementary Table S2 details the variables included in the analysis in conjunction with their definitions and data sources.

### Geocoding

Extracted STH survey data were geolocated to latitude and longitude coordinates. Location data at the lowest available administrative level were obtained from Google Earth. Where STH prevalence survey data were reported at a village level or district level, coordinates of the centroid were used for georeferencing. The survey locations for each study were stored in the geographical information system ArcGIS (ESRI, Redlands, California, USA). To create the spatially referenced dataset for analysis, data on STH prevalence and covariates were linked according to location using ArcGIS Pro, which was also used to produce the visualizations, including the geospatial prediction maps.

### Geospatial analysis

Bayesian model-based geostatistics (MBG) was used to predict the prevalence of each STH species mapped to a 1 km^2^ resolution. In the Bayesian binomial geostatistical analysis, the number of infected people among those surveyed was used as the outcome, and fixed-effect covariates and spatial random effects were included in the model.^26^ Covariates for the final spatial model were selected using a fixed-effects logistic regression model with an exclusion criterion of Wald (p > 0.2). To avoid collinearity, covariates that were highly correlated (variance inflation factor (VIF) > 4) were excluded from the model. For each STH species, an independent geospatial model was constructed. The proportion of cases at each surveyed location *j* was assumed to follow a binomial distribution: $Y_{j}∼\;Binomial\;\left(n_{j},p_{j}\right)$; where $Y_{j}$ was the observed prevalence of infection, $n_{j}$ was the number of participants screened for infection and $p_{j}$ was the predicted prevalence at location j, with j=(1,…,n). The predicted prevalence was associated via a logit link function to a linear combination of predictors defined as:

$$\text{logit}\left(p_{j}\right)=\log\left(\frac{p_{j}}{1\;-\;p_{j}}\right)=\alpha +\sum_{z=1}^{Z}\beta_{z}X_{z,j}\;+\;\zeta_{j\;}$$

where *α* is the intercept, *β* is a matrix of covariate coefficients, X is a matrix of Z covariates, and $\zeta_{j\;}$ is a spatial random effect, which was represented by a zero-mean stationary Gaussian process with a Matérn covariance function. The covariance function was defined by two parameters: the range ρ, which represents the distance beyond which correlation becomes negligible (∼0.1), and σ the marginal standard deviation.^47^^,^^48^ We used the shape parameter value of 1 to control the smoothness of the spatial surface. Due to the Bayesian characteristics of the geospatial model, priors were defined for all parameters (and hyperparameters) in the model. Non-informative priors were used for *α* (uniform prior with bounds –∞ and ∞) and normal priors were set with mean = 0 and precision (the inverse of the variance) = 1 × 10^−4^ for each $\beta_{z}$. The default priors were used for the parameters of the spatial random field.^49^ We used integrated nested Laplace approximations approach in R statistical software (R-INLA) and the stochastic partial differential equations approach to do fast approximate Bayesian inference.^47^^,^^48^ To ensure a satisfactory characterization of the posterior distribution of all parameters, a relatively large number of samples (10,000 samples) was computed. The best fitting model was selected using Watanabe–Akaike information criterion (WAIC). The models were validated using INLA's probability integral transform (PIT) and conditional predictive ordinates (CPO) statistics. From our geospatial analysis, we obtained maps for the predicted prevalence of each STH infection at pixel level (1 km^2^). The posterior mean, standard deviation, and 95% credible intervals (CrI) were estimated for all the parameters.

The data were categorised into two periods (1998–2011 and 2012–2021) to assess changes in the prevalence of STH, taking into account the midpoint of publication dates for included studies, the launch of the WHO NTD Roadmap in 2012,^50^ and the concurrent signing of the London Declaration on NTDs, which represented a landmark commitment by pharmaceutical companies to large-scale drug donations, significantly accelerating global control efforts. Predicted prevalence maps for each STH species at both periods were produced.

### Ethics approval

Not applicable as this analysis is based on published work.

### Consent to participate

Not applicable as this review is based on published work.

### Role of the funding source

The funders had no role in the study design, decision to publish, or preparation of the manuscript.

## Results

**Fig. 1:**
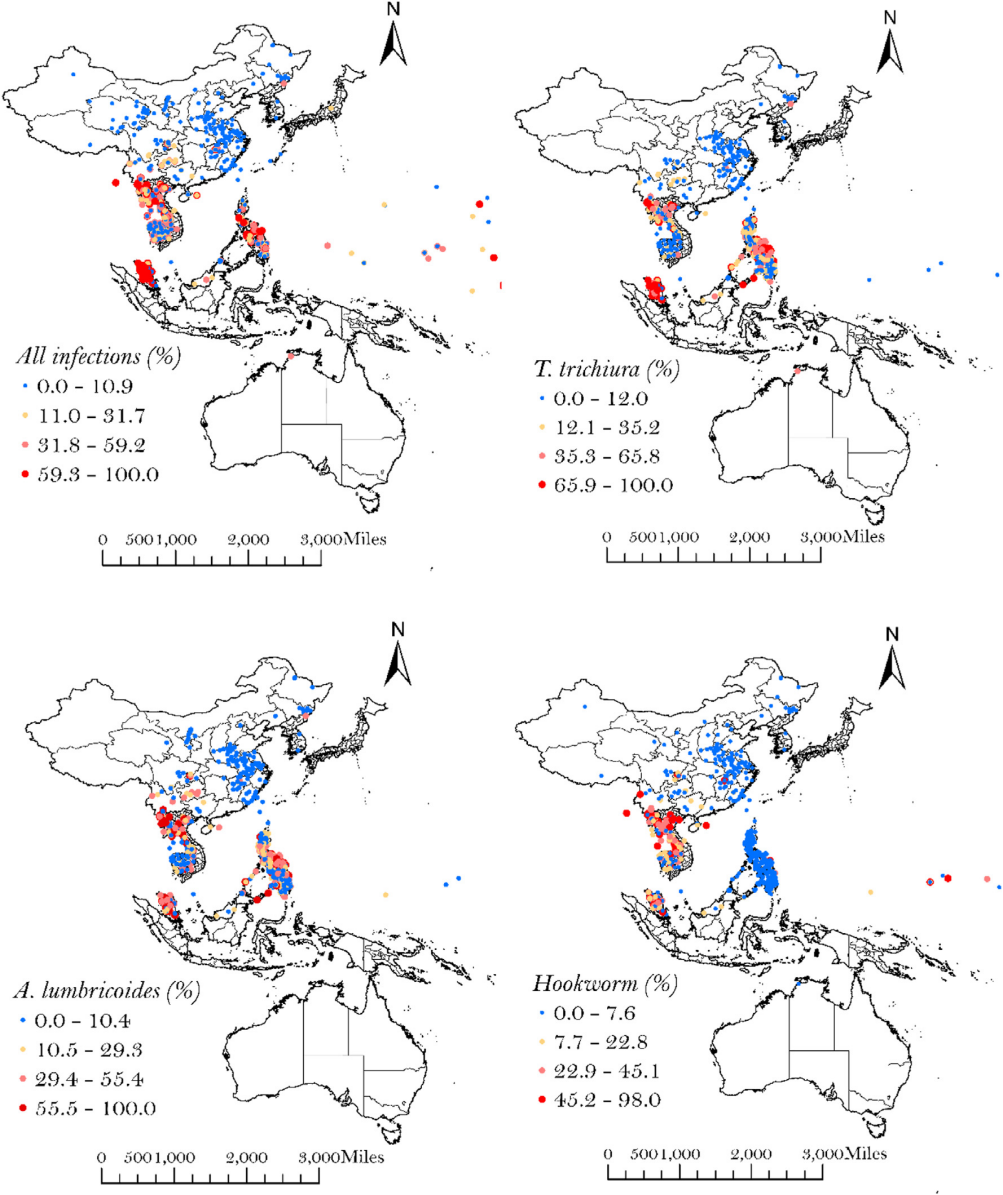

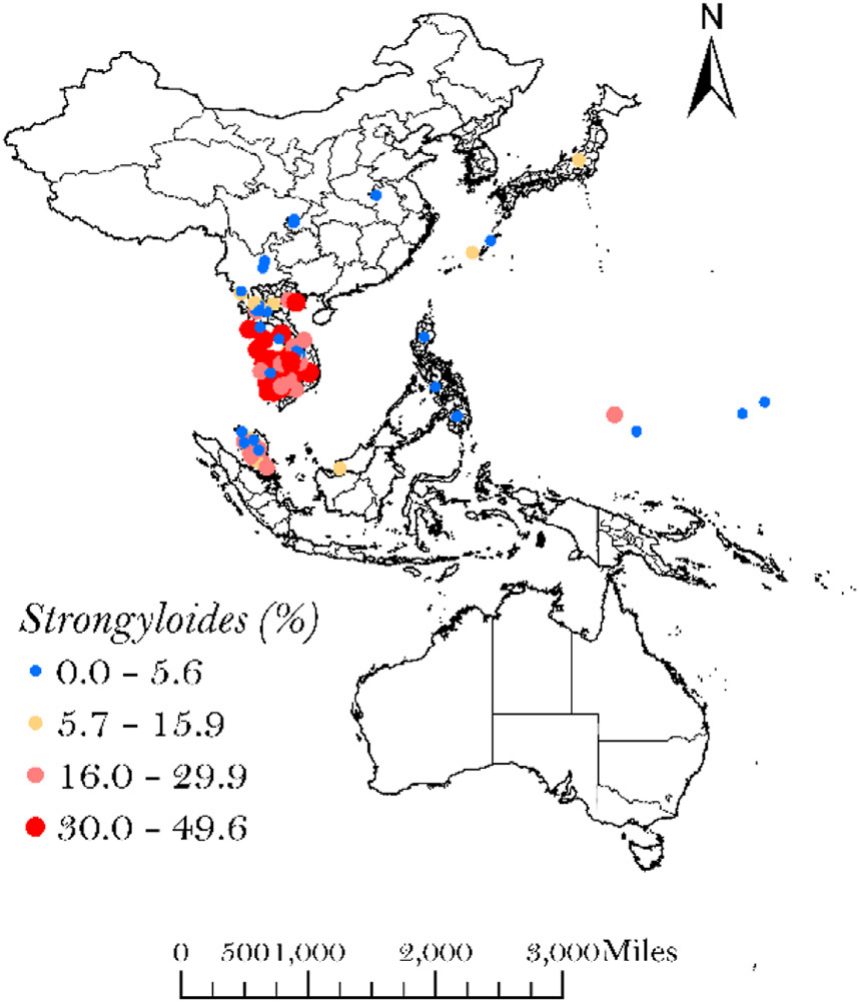
Survey locations with STH prevalence (%) within the WPR from 1998 to 2021.

Data were extracted from 227 prevalence surveys published between 2000 and 2023, representing data collection undertaken between 1998 and 2021. The study selection process is presented in the PRISMA flowchart (Supplementary Figure S1 (the PRISMA checklist is detailed in Supplementary Table S3)), and detailed characteristics of the included studies are presented in Supplementary Table S4. Survey data were available for Australia, Cambodia, China, Fiji, Japan, Laos, Malaysia, Marshall Islands, Philippines, Papua New Guinea (PNG), Republic of Korea, Solomon Islands, Tonga, Tuvalu and Vietnam. Of the 227 studies included in the analysis, 62 undertook prevalence surveys in indigenous/ethnic minority populations. The 227 studies represented 3122 unique survey data points which were included in the analysis. On the basis of the data point locations, Fig. 1 shows observed STH prevalence across the WPR.

### STH prevalence in the WPR

The STH prevalence estimates for countries within the region are detailed in Table 1.

**Table 1:** Pooled prevalence estimates at the country level for each species of STH infection in the WPR.

Country	All infections		Hookworm		*Strongyloides*		*T. trichiura*		*A. lumbricoides*	
	Prevalence (95% CI)	Data points (n)	Prevalence (95% CI)	Data points (n)	Prevalence (95% CI)	Data points (n)	Prevalence (95% CI)	Data points (n)	Prevalence (95% CI)	Data points (n)
Vietnam	44.5 (33.0, 56.7)	59	26.8 (13.4, 46.3)	18	13.4 (6.5, 25.7)	8	26.6 (14.5, 43.8)	16	25.8 (12.8, 45.0)	17
Malaysia	41.5 (35.9, 47.4)	303	11.3 (8.8, 14.5)	97	9.7 (6.5, 14.4)	23	44.7 (32.9, 57.1)	83	27.1 (20.0, 35.6)	81
Laos	32.6 (27.5, 38.1)	290	28.8 (23.2, 35.1)	87	6.7 (3.9, 11.4)	38	12.1 (8.7, 16.6)	82	11.3 (7.3, 17.2)	81
Philippines	29.6 (27.2, 32.2)	1653	10.7 (9.0, 11.2)	539	0.9 (0.3, 2.5)	7	14.2 (12.5, 16.0)	550	13.4 (12.0, 14.9)	553
Solomon Isl.	28.4 (9.7, 59.4)	22	14.2 (2.4, 53.1)	7	5.7 (2.3, 13.3)	3	8.2 (1.6, 32.8)	3	1.8 (1.0, 26.4)	3
Cambodia	24.4 (18.3, 31.7)	175	13.1 (10.1, 16.9)	67	23.9 (13.2, 39.6)	11	2.1 (1.3, 3.3)	49	16.1 (7.8, 30.2)	54
PNG	8.6 (3.5, 19.6)	5	NA		8.7 (1.9, 31.9)	2	NA		NA	
Japan	6.4 (3.3, 12.0)	3	NA		6.4 (3.3, 12.0)	3	NA		NA	
China	4.3 (3.2, 5.8)	606	3.2 (2.1, 4.9)	310	0.04 (0, 2.1)	8	6.5 (3.4, 12.1)	173	6.9 (3.8, 12.1)	188
Republic of Korea	0.02 (0, 0.12)	6	0	2	NA		0.05 (0.01, 0.4)	2	0	2

Key: NA: Countries with no data to pool estimates. All infections are the sum of each STH species and include studies that did not differentiate the STH species. Hookworm: *A. duodenale*, *N. americanus*, *A. ceylanicum* and *Ancylostoma* spp were classified collectively as hookworm. *Strongyloides*: *S. stercoralis* and strongylodiasis were classified collectively as *Strongyloides*. *T. trichiura*: Trichuris and trichuriasis were included as *T. trichiura*. *A. lumbricoides:* Ascaris was included as *A. lumbricoides*.

For ‘all infections’ (defined as the sum of all species and inclusive of studies that did not differentiate the STH species), six countries in the region were estimated to have an infection prevalence >20%: Vietnam 44.5% (95% CI: 33.0, 56.7); Malaysia 41.5% (95% CI: 35.9, 47.4); Laos 32.6% (95% CI: 27.5, 38.1); Philippines 29.6% (95% CI: 27.2, 32.2); Solomon Islands 28.4% (95% CI: 9.7, 59.4) and Cambodia 24.4% (95% CI: 18.3, 31.7). Of the different STH species, the highest prevalence of hookworm was estimated to occur in Laos at 28.8% (95% CI: 23.2, 35.1) followed by Vietnam 26.8% (95% CI: 13.4, 46.3) and the highest prevalence of *Strongyloides* was estimated to occur in Cambodia 23.9% (95% CI: 13.2, 39.6) followed by Vietnam 13.4% (95% CI: 6.5, 25.7). *T. trichiura* infection prevalence was high in Malaysia at 44.7% (95% 32.9, 57.1) and Vietnam at 26.6% (95% CI: 14.5, 43.8). *A. lumbricoides* infection prevalence was estimated to be high in Malaysia at 27.1% (95% CI: 20.0, 35.6) and Vietnam at 25.8% (95% CI: 12.8, 45.0). Results for Australia are not reported in the above table due to the low number of available data points. Across five data points, the pooled prevalence for all infections in Australia was 10.6% (95% CI: 3.2, 30.0) and across two data points, the pooled prevalence of *T. trichiura* infection was 20.7% (95% CI: 3.9, 62.7).

Infection prevalence in the WPR over time has shown a reduction from 26.7% (95% CI: 22.2, 25.2) in 1998–2011 to 6.3% (95% CI: 6.2, 6.4) in 2012–2021 (Table 2). The trend in STH prevalence by species across the two reporting periods showed a relative reduction of 82.6%, 56.9% and 70% in hookworm, *T. trichiura* and *A. lumbricoides* infection respectively, but conversely there was a 38.3% increase in *Strongyloides* prevalence (Table 2).

**Table 2:** Pooled prevalence estimates at the country level for each species of STH in the WPR categorized by survey period.

Country	Survey period	Pooled infection prevalence % (95% CI)				
		All infections	Hookworm	*Strongyloides*	*T.trichiura*	*A. lumbricoides*
Vietnam	Old	38 (30.3, 45.8)	26.8 (13.4, 46.3)	NA	26.6 (14.5, 43.8)	25.8 (12.8, 45.0)
	Recent	17.3 (7.0, 27.6)	NA	17.3 ((7.0, 27.6)	NA	NA
Malaysia	Old	49.5 (34.4, 64.5)	20.6 (17.4, 23.8)	NA	62.4 (42.8, 82.1)	41.2 (25.3, 57.1)
	Recent	36 (31.0, 41.0)	13.4 (6.6, 20.2)	9.7 (6.5, 14.4)	55.7 (43.3, 68.8)	20.1 (15.0, 35.6)
Laos	Old	37.9 (23.3, 48.5)	39.8 (33.4, 46.3)	5.8 (3.8, 7.8)	22 (17.7, 26.3)	27.6 (22.6, 32.6)
	Recent	20.1 (18.0, 22.3)	23.8 (18.4, 46.3)	24.8 (17.9, 31.6)	8.0 (6.1, 9.9)	5.4 (3.9, 6.8)
Philippines	Old	20.7 (17.7, 23.7)	2.9 (1.9, 3.9)	1.2 (0.5, 2.8)	34.8 (25.0, 44.5)	24.4 (17.6, 31.3)
	Recent	19.3 (18.2, 20.4)	5.4 (3.7, 7.1)	1.8 (0.8, 4.4)	22.1 (19.7, 24.6)	10.2 (8.5, 21.8)
Solomon Isl.	Old	NA	NA	NA	NA	NA
	Recent	28.4 (9.7, 59.4)	14.2 (2.4, 53.1)	5.7 (2.3, 13.3)	8.2 (1.6, 32.8)	1.8 (1.0, 26.4)
Cambodia	Old	11 (9.9, 12.1)	15.8 (13.1, 26.5)	22 (5.7, 38.2)	2.8 (2.0, 3.6)	4.7 (3.5, 5.8)
	Recent	13.7 (12.1, 15.3)	10.1 (8.9, 20.3)	35.5 (28.0, 43.1)	0.3 (0.2, 0.6)	0.4 (0.2, 1.3)
PNG	Old	NA	NA	NA	NA	NA
	Recent	8.6 (3.5, 19.6)	NA	8.7 (1.9, 31.9)	NA	NA
Japan	Old	7.2 (2.0, 12.3)	NA	NA	NA	NA
	Recent	NA	NA	NA	NA	NA
China	Old	15.7 (14.7, 16.7)	17.5 (15.8, 19.1)	NA	15 (12.9, 17.1)	16.8 (13.9, 19.8)
	Recent	0.4 (0.3, 0.5)	0.9 (0.8, 1.0)	0.04 (0, 2.1)	0.1 (0.08, 0.3)	0.3 (0.2, 0.6)
Korea, Republic of	Old	NA	NA	NA	NA	NA
	Recent	0.02 (0, 0.12)	NA	NA	NA	NA
**Overall pooled prevalence**	Old	26.7 (22.2, 25.2)	21.3 (19.7, 22.8)	13.3 (7.5, 19.1)	22.5 (20.2, 25.1)	21.7 (20.4, 23.1)
	Recent	6.3 (6.2, 6.4)	3.7 (3.6, 3.9)	18.4 (14.9, 21.9)	9.7 (9.5, 9.9)	6.5 (6.3, 6.7)
	P-value	0.0001	0.001	0.001	0.000	0.002

Key: Old survey period relates to the period between 1998 and 2011. Recent survey period relates to the period between 2012 and 2021.

Over the total study period, on the basis of population group surveyed, indigenous/ethnic minority populations recorded the highest prevalence of *A. lumbricoides* (47.7%; 95% CI: 37.6, 58.0) hookworm (29.0%: 95% CI: 19.2, 41.2) and *T. trichuira* (59.6%; 95% CI: 49.9, 68.6) infection and community surveys recorded the highest prevalence of *Strongyloide*s infection (19.2%: 95% CI: 8.7, 63.9) Table 3.

**Table 3:** Overall pooled prevalence of STH infections for different population groups in the WPR, 1998–2021.

Population	Pooled prevalence of infection % (95% CI)							
	Hookworm	Data points	*Strongyloides*	Data points	*T. trichiura*	Data points	*A. lumbricoides*	Data points
Children	16.7 (8.9, 29.2)	509	16.6 (5.0, 43.0)	11	25.0 (14.7, 39.2)	521	27.8 (19.1, 38.6)	521
Community	19.2 (6.2, 46.2)	95	19.2 (8.7, 63.9)	19	19.2 (6.2, 46.2)	90	38.1 (13.5, 70.8)	89
Ethnic minority	29.0 (19.2, 41.2)	47	16.2 (8.7, 28.2)	17	59.6 (49.9, 68.6)	44	47.7 (37.6, 58.0)	44

Key: CI: confidence interval. Population groups were classified as: children-the age to define a child was determined by the study and included pre-SAC and SAC; community surveys were undertaken across all age groups; the ethnic minority classification if appropriate took precedence over age (i.e., children vs. community).

Studies included in the analysis had an average QA assessment score of 6 indicating ‘medium’ quality. The scoring was based on the modified Newcastle-Ottawa Quality Assessment Scale,^37^ with full details provided in Supplementary Table S5.

### Spatial prediction of STH infections

**Fig. 2:**
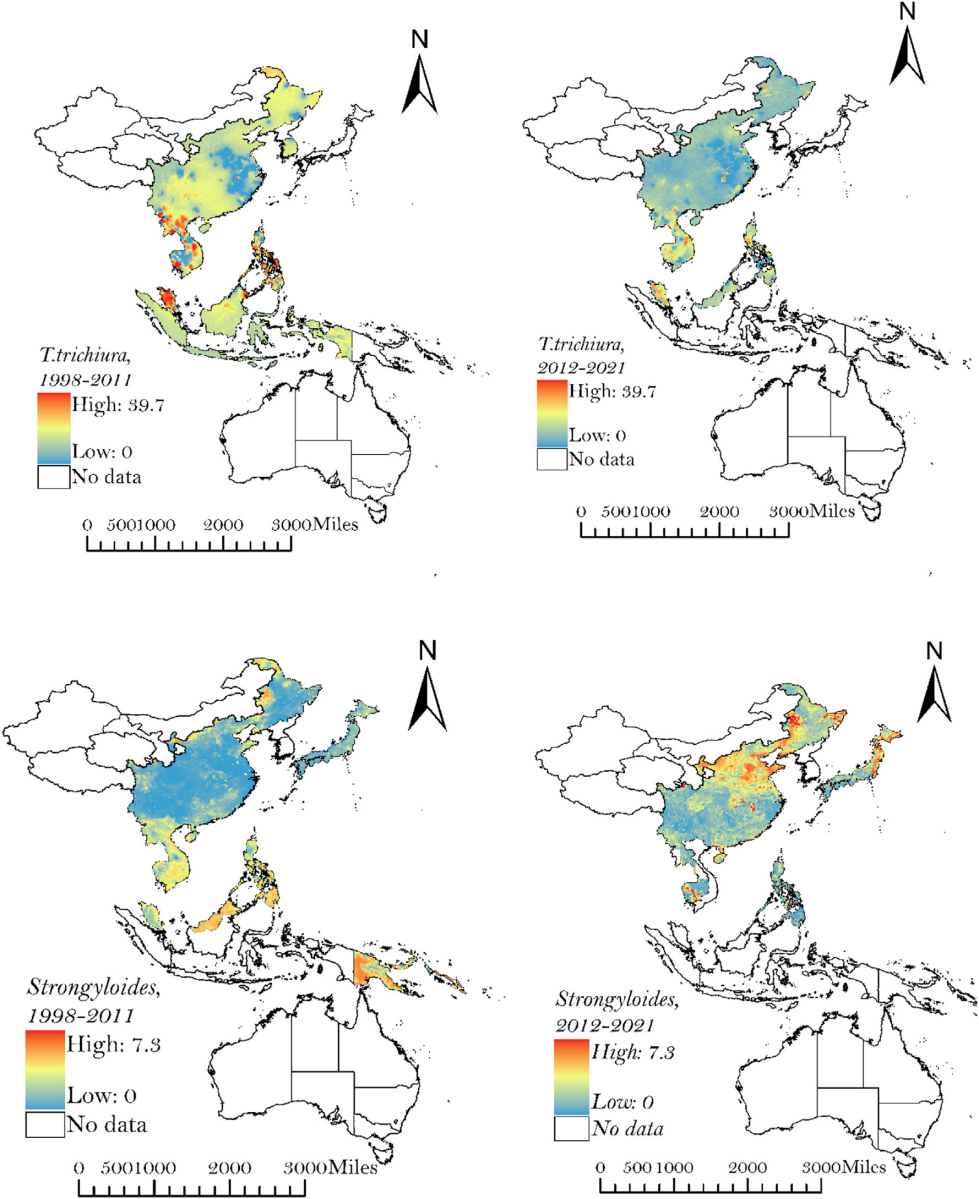

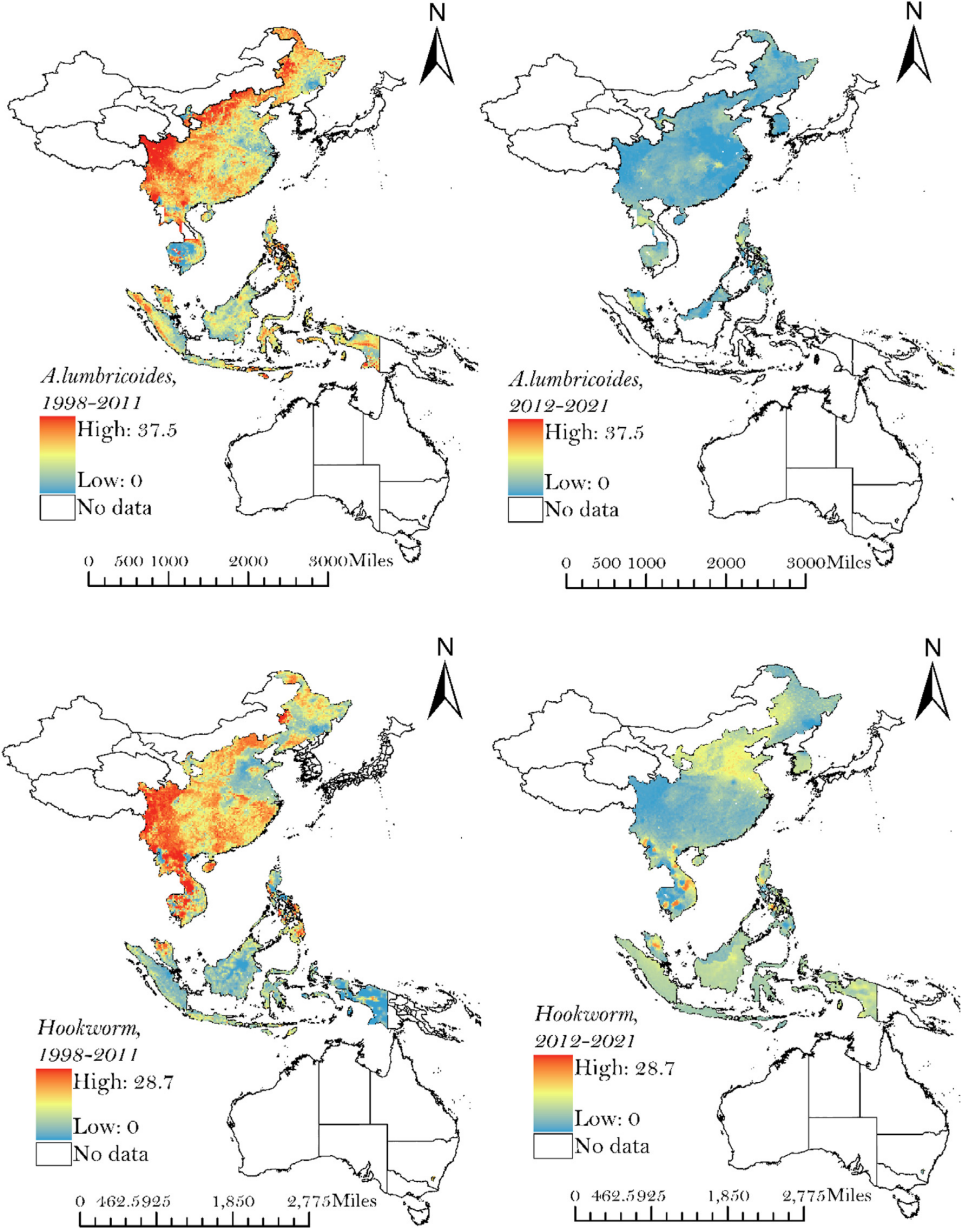
Predicted geographical distribution of STH infections for 1998–2011 and 2012–2021 across the WPR.

Our results provide estimates of hookworm, *Strongyloides, T. trichiura,* and *A. lumbricoides* prevalence across the WPR at a resolution of 1 km^2^. Fig. 2 illustrates the estimates of infection for each STH species, for 1998 to 2011 and 2012 to 2021. The maps show substantial spatial variation in the predicted distribution of STH prevalence between and within countries in the WPR, in addition to highlighting the countries for which there is no data. The maps show the reduction and change in predicted infection prevalence over time for each STH species.

Over the last reporting period, 2012–2021, foci with the highest predicted prevalence of hookworm occurred in central, southern and eastern regions of China, Cambodia and Malaysia. Northern regions of China had the highest predicted prevalence of *Strongyolides* and Australia had the highest predicted prevalence of *T. trichiura.* High levels of *A. lumbricoides* prevalence were predicted in northwest China, northern Cambodia and Malaysia.

The prediction standard deviation plots for each STH species are detailed in Supplementary Figure S2.

Due to the limited availability of data across large geographies, Australia and Western China are excluded from the maps in Fig. 2. Noting the limitations of data availability, predictive maps including Australia and Western China are included in Supplementary Figure S3.

The predicted geographical distribution for each STH species, based on 1998–2021 data, at administrative levels across the WPR are detailed in Supplementary Figure S4.

### Factors associated with the prevalence of STH infection

Each STH species had their own unique combination of covariates associated with the best geospatial model to predict infection prevalence. The VIF results for variables evaluated to inform the best fit model for each STH species are presented in Supplementary Table S6. Odds ratios with 95% credible intervals (CrI) for all covariates with a VIF <4 is detailed in Table 4.

**Table 4:** Odds ratios with 95% credible intervals (CrI) for all covariates prior to exclusion using WAIC.

Variables	Odds ratio (95% CrI)			
	Hookworm	*Strongyloides*	*T. trichiura*	*A. lumbricoides*
Altitude (km)	**5.31 (3.03, 9.29)**	*NA*	0.88 (0.58, 1.34)	0.57 (0.36, 1.09)
Distance to health facility (walking time in minutes)	0.52 (0.36, 1.34)	**7.81 (3.90, 16.9)**	**3.21 (2.12, 4.6)**	**7.18 (4.71, 10.91)**
Distance to water body (km)	0.82 (0.73, 0.91)	0.75 (0.42, 1.34)	1.05 (0.97, 1.15)	**0.76 (0.70, 0.84)**
Population density (person/km^2^)	0.99 (0.99, 1.01)	1.05 (0.99, 1.11)	**0.90 (0.89, 0.92)**	0.99 (0.73, 1.01)
Solar radiation (kJ m-2 day-1)	0.24 (0.21, 4.74)	*NA*	6.74 (0.34, 15.73)	**0.47 (0.33, 0.67)**
Volume fraction of coarse fragments (>2 mm; %)	**0.62 (0.47, 0.82)**	0.50 (0.22, 1.15)	**0.08 (0.06, 0.10)**	**0.27 (0.21, 0.35)**
Bulk density of the fine earth fraction (cg/cm^3^)	1.14 (0.96, 1.35)	1.20 (0.22, 2.15)	1.27 (0.92, 1.49)	0.84 (0.73, 1.03)
Nitrogen (cg/kg^1^)	**0.27 (0.18, 0.42)**	*NA*	**1.42 (1.05, 1.94)**	1.28 (0.98, 1.69)
Sand (>0.05 mm) in fine earth (%)	**5.11 (3.98, 6.61)**	**3.63 (1.80, 7.54)**	**2.06 (1.52, 2.88)**	**2.38 (1.95, 2.89)**
Silt (0.002–0.05 mm) in fine earth (%)	*NA*	**0.34 (0.21, 0.21)**	*NA*	*NA*
Organic carbon in fine earth (hg/m^3^)	1.39 (0.99, 1.92)	*NA*	**0.68 (0.54, 0.85)**	**0.51 (0.40, 0.61)**
Wetland cover (herbaceous vegetation ≥10% and permanently/regularly flooded)	1.03 (0.99, 1.97)	7.53 (0.52, 106.31)	**1.18 (1.12, 1.23)**	**1.20 (1.14, 1.25)**
Grass cover (≥10% herbaceous plant cover)	*NA*	*NA*	*NA*	1.15 (0.95, 1.39)
Bare land cover (≤10% vegetated cover)	*NA*	1.86 (0.53, 6.58)	**0.09 (0.05, 0.16)**	*NA*

Key*: NA:* Variables excluded from the model due to high collinearity (VIF >4); Bold: variables associated with the outcome; CrI: credible interval; WAIC: Watanabe–Akaike information criterion.

Variables that were found to be associated with the outcome included a positive association between altitude (km) and hookworm prevalence (5.31, 95% CrI: 3.03, 9.29). This means that the odds of hookworm infection increased by 5.31 times for every unit increase in altitude.

The distance to a health facility (walking time in minutes) showed a strong positive association with *A. lumbricoides* (7.18, 95% CrI: 4.71, 10.91). Sand content (>0.05 mm, %) in the soil was positively associated with the prevalence of all four STH species, with coefficients of 5.11 (95% CrI: 3.98, 6.61) for hookworm, 3.63 (95% CrI: 1.80, 7.54) for *Strongyloides*, 2.06 (95% CrI: 1.52, 2.88) for *T. trichiura*, and 2.38 (95% CrI: 1.95, 2.89) for *A. lumbricoides.* The volume fraction of coarse soil fragments (>2 mm; %) and organic carbon content in fine earth (hg/m^3^) were negatively associated with the prevalence of *T. trichiura* (0.08, 95% CrI: 0.06, 0.10; 0.68, 95% CrI: 0.54, 0.85) and *A. lumbricoides* (0.27, 95% CrI: 0.21, 0.35; 0.51, 95% CrI: 0.40, 0.61). Silt content (0.002–0.05 mm, %) was negatively associated with *Strongyloides* prevalence (0.34, 95% CrI: 0.21, 0.56), while soil nitrogen content (cg/kg) was positively associated with *T. trichiura* prevalence (1.42, 95% CrI: 1.05, 1.94). *T. trichiura* prevalence was positively associated with wetland cover (herbaceous vegetation ≥10% and permanently/regularly flooded) (1.18, 95% CrI: 1.12, 1.23) and negatively associated with bare land cover (≤10% vegetation cover) (0.09, 95% CrI: 0.05, 0.16).

The Watanabe–Akaike information criterion results to select the best fitting models are detailed in Supplementary Table S7 and are summarized in Supplementary Table S8. The results of the posterior predictive methods (CPO and PIT) that were used to measure the predictive ability of the models are summarized as Diagnostic Plots in Supplementary Figure S5. The model comparison, using Deviance Information Criterion (DIC), that justifies setting the spatial correlation between separate landmasses to zero is provided in Supplementary Figure S6. Supplementary Table S9 provides a comparison between the predicted prevalence (aggregated at the country level) and the pooled prevalence estimates from the meta-analysis for each STH species.

## Discussion

Understanding STH epidemiology is a priority for the WPR, which has been shown to carry the highest regional burden of infection amongst its school-age population.^51^ The WHO classify the WPR as a high risk zone for STH infection,^9^ and in view of the resource implications of undertaking prevalence surveys, seeking data alternatives to inform public health policy has become a priority.^12^ Analyses based on geostatistical modelling provide a robust approach to NTD risk profiling across different geographical scales,^29^ and to our knowledge, this is the first to be undertaken for STH infections across the WPR. Using a geospatial modelling approach, this study generated the first high-resolution maps of STH prevalence for each species across the WPR. The findings revealed substantial national and local variation in STH prevalence. Our study also identified several environmental factors influencing the spatial distribution of each STH species in the region.

There were substantial variations in prevalence estimates of STH infection between countries and across time periods. Studies that have investigated the geospatial distribution of STH infections in other regions of the world have found comparable variations between countries.^29^ These findings provide important information to inform the prioritization of regional STH control program resources. Results showed infection prevalence has reduced over time for most STH species with the exception of *Strongyloides*, for which increased prevalence has been attributed to ineffective PC regimes, advances in diagnostic techniques, increased global migration and the rising use of immunosuppressant medications.^6^^,^^52^, ^53^, ^54^ The auto-infective capacity of *Strongyloides* means that infection can last for the host's lifetime and imported strongyloidiasis into non-endemic countries is being increasingly reported.^55^^,^^56^ Although transient and migrant populations were excluded from our analysis, the auto-infective capacity of *Strongyloides*, may account for the infections identified in Japan, which is one of the countries in the region claiming to have eliminated STHs.^57^

Strongyloidiasis is one of the most neglected of the NTDs, which is reflected in its exclusion from the classification of parasites that relate to soil-transmitted helminthiases in the 2012–2020 NTD roadmap.^12^ The recent publication of preventative chemotherapy guidelines to control strongyloidiasis,^58^ satisfies one of the targets in the 2021–2030 NTD roadmap which includes *S. stercoralis* within its STH parasite classification.^12^ The high resolution prevalence maps produced from this study will also contribute to a 2021–2030 NTD roadmap target, by improving understanding of *S. stercoralis* epidemiology.^12^

The reduction in ‘all infections’, hookworm, *T. trichiura* and *A. lumbricoides* prevalence across the WPR over time may in-part, reflect improvements in socio-economic development and be the results of actions implemented through the NTD roadmaps and the Regional Action Plan for Neglected Tropical Diseases in the Western Pacific.^12^^,^^50^^,^^59^ The analysis however highlights the fact that these infections persist in all countries across the region and that they are not solely correlated with economic development. STH infections remain endemic in indigenous populations of Australia, despite the country's high Human Development Index (HDI) ranking.^60^^,^^61^

Although the WHO's primary strategy to overcome the STH epidemic is the periodic treatment of at-risk populations living in endemic areas,^1^ experimental and modelling studies suggest that community-wide treatment may be more effective compared to chemotherapy in these groups alone.^17^^,^^18^ Interestingly, this study found infection prevalence to be higher in the community than in children across all four STH species, although it is noted that the community category would have also included children. In 2022 the WHO estimated that 68 million pre-SAC and SAC required PC within the following countries of the WPR- Cambodia, Fiji, Kiribati, Laos, Marshall Islands, Micronesia, Nauru, PNG, Philippines, Solomon Islands, Tonga, Tuvalu and Vietnam.^4^ Although we did not obtain sufficient data to undertake an analysis for a number of these countries, our results did identify a high prevalence estimate for Malaysia which was omitted from the WHO 2022 listing of countries recommended to undertake PC in pre-SAC and SAC. Although the WHO 2022 listing indicates that no data were available for 12.9% of the WPR,^4^ our study identified a large number of data points for Malaysia. Acknowledging that the omission of Malaysia from the WHO listing could relate to timeframes, surveys undertaken in Malaysia between 2020 and 2022 still yielded high prevalence figures, especially within indigenous ethnic minorities (data not shown) raising the question as to whether other vulnerable population groups should also be prioritized in intervention strategies.

This study provided an opportunity to predict infection prevalence where no survey data were available and by exhaustive fitting of all possible Bayesian geostatistical models, one was identified to predict infection prevalence for each STH species. The free living-stages of STH species are susceptible to environmental factors,^62^ and our analysis showed different variables to be associated with the best fitting model for different STH species. In accordance with other studies, our modelling showed altitude to be associated with the prevalence of *A. lumbricoides*, *T. trichuris* and hookworm,^63^ but not *Strongyloides*.^64^ Likewise, other studies have found soil characteristics such as sand and nitrogen content to be of significance,^65^^,^^66^ while pH was not.^66^ Our modelling did not identify precipitation as an associated variable and although other studies have found moisture to be of significance, their results evaluated temporal differences.^66^^,^^67^ The findings of this study demonstrate the relationship between different environmental and ecological variables and STH prevalence and although all countries in the region showed some risk of infection, of note are the regions of high predicted risk for *Strongyloides* in PNG and areas of China. Similarly, high-risk areas in China, PNG and Australia were predicted for *T. trichiuris* for which survey data are not currently available. Although data were not available at the required level of resolution to consider additional variables that have also been associated with STH prevalence (e.g., access to clean water, sanitation and hygiene, socio-economic status^1^) this study provides an important baseline to identify vulnerable populations for screening within high-risk areas.

Although these results provide a valuable risk assessment baseline, they need to be considered in the context of the following limitations. Data challenges present in terms of availability, quality, and acquisition methodology as surveys lack the sophistication and rigor associated with other types of studies such as clinical trials.^68^^,^^69^ Data availability was uneven across countries, with some areas lacking recent data, leading to potential gaps in the analysis. Diagnostic methods and sampling techniques are not universal across all surveys, and this has the potential to affect the consistency of the prevalence estimates. There is a possibility of survey data bias, as survey locations or populations may have been chosen on the basis of a high risk of infection, thereby potentially overestimating the burden of STH infection. The prevalence estimates, from surveys undertaken using the WHO's sampling design, which aggregates data to larger areas, may underestimate disease prevalence in high-risk hotspots.^70^ The consideration and timeframes of PC programs are not factored into the analyses. The exclusion of some covariates due to multicollinearity or data limitations might have constrained the study's ability to explain the spatial distribution of infections. While the study provides detailed maps for the WPR, the findings may not be directly applicable to other regions with different contexts.

Some of these limitations can be viewed as future research opportunities, if HDI metrics were available at a high level of resolution, many areas of health research have the potential to benefit. The study highlights the countries where survey data are unavailable or extremely scarce and that would benefit from survey data to inform future modelling, noting that model based estimates should be iteratively updated.^71^

### Conclusions

This study provides important information on high-risk areas for STH infections in the WPR which can be used to prioritize the identification of vulnerable populations in the region. The analytical methods employed in this study maximize the impact of available data as large-scale surveys are often cost prohibitive. The results of our study contribute to the WHO objective of STH mapping to identify areas of risk, and the modelling also builds on geospatial analyses techniques and their contribution to NTD elimination.

## Contributors

BG, KAA and AC conceived the study. KAA led the overall activities of the manuscript. BG and TT undertook the systematic search and screening of the studies. BG, HFW, AML and TYA contributed to the data cleaning. HFW, BG and KAA had full access to and verified the underlying data. HFW and KAA led and verified the analysis which was undertaken by HFW. BG and KAA drafted the manuscript, and all authors critically revised the manuscript for methodological and intellectual content. All authors have read and approved the final manuscript, with BG, HFW and KAA responsible for the decision to submit.

## Data sharing statement

Relevant data is included within the manuscript and supporting documentation.

## Editor note

The Lancet Group takes a neutral position with respect to territorial claims in published maps and institutional affiliations.

## Declaration of interests

All authors declare that they have no conflicts of interest.

## References

[1] World Health Organization (2023). Soil-transmitted helminth infections key facts. https://www.who.int/news-room/fact-sheets/detail/soil-transmitted-helminth-infections.

[2] Centres for Disease Control and Prevention (2022). Parasites- Soil-transmitted helminths. https://www.cdc.gov/parasites/sth/index.html.

[3] Global Atlas of Helminth Infections (2023). Global burden. https://www.thiswormyworld.org/worms/global-burden.

[4] World Health Organization (2023). The global health observatory soil-transmitted helminthiasis. https://www.who.int/data/gho/data/themes/topics/soil-transmitted-helminthiases.

[5] Montresor A., Mupfasoni D., Mikhailov A. (2020). The global progress of soil-transmitted helminthiases control in 2020 and World Health Organization targets for 2030. PLoS Negl Trop Dis.

[6] World Health Organization (2023). Control of neglected tropical diseases strongloidiasis key facts. https://www.who.int/teams/control-of-neglected-tropical-diseases/soil-transmitted-helminthiases/strongyloidiasis.

[7] Beknazarova M., Whiley H., Ross K. (2016). Strongyloidiasis: a disease of socioeconomic disadvantage. Int J Environ Res Public Health.

[8] Page W., Judd J.A., Bradbury R.S. (2018). The unique life cycle of strongyloides stercoralis and implications for public health action. Trop Med Infect Dis.

[9] World Health Organization (2011).

[10] Campbell S.J., Nery S.V., Doi S.A. (2016). Complexities and perplexities: a critical appraisal of the evidence for soil-transmitted helminth infection-related morbidity. PLoS Negl Trop Dis.

[11] Pabalan N., Singian E., Tabangay L., Jarjanazi H., Boivin M.J., Ezeamama A.E. (2018). Soil-transmitted helminth infection, loss of education and cognitive impairment in school-aged children: a systematic review and meta-analysis. PLoS Negl Trop Dis.

[12] World Health Organization (2020).

[13] World Health Organization (2023).

[14] World Health Organization Control of neglected tropical diseases- control strategy soil-transmitted helminthiases. n.d.A. https://www.who.int/teams/control-of-neglected-tropical-diseases/soil-transmitted-helminthiases/control-strategy.

[15] Specht S., Keiser J. (2023). Helminth infections: enabling the world health organization road map. Int J Parasitol.

[16] Means A.R., Werkman M., Walson J.L. (2017). Prospects for elimination of soil-transmitted helminths. Curr Opin Infect Dis.

[17] Dyer C.E., Ng-Nguyen D., Clarke N.E. (2023). Community-wide versus school-based targeted deworming for soil-transmitted helminth control in school-aged children in Vietnam: the CoDe-STH cluster-randomised controlled trial. Lancet Reg Health West Pac.

[18] Pullan R.L., Halliday K.E., Oswald W.E. (2019). Effects, equity, and cost of school-based and community-wide treatment strategies for soil-transmitted helminths in Kenya: a cluster-randomised controlled trial. Lancet.

[19] World Health Organization The global health observatory- reported number of people requiring interventions against NTDs. n.d.B. https://www.who.int/data/gho/data/indicators/indicator-details/GHO/reported-number-of-people-requiring-interventions-against-ntds.

[20] World Health Organization (2022). Accelerating work to overcome the global impact of NTDs: 2011-2020 progress dashboard. https://www.who.int/teams/control-of-neglected-tropical-diseases/overview/progress-dashboard-2011-2020.

[21] World Health Organization Control of negelected tropical disease- PCT databank- soil-transmitted helminthiases. n.d.C. https://www.who.int/teams/control-of-neglected-tropical-diseases/data-platforms/pct-databank/soil-transmitted-helminthiases.

[22] Dwyer-Lindgren L., Cork M.A., Sligar A. (2019). Mapping HIV prevalence in sub-Saharan Africa between 2000 and 2017. Nature.

[23] Alene K.A., Wagaw Z.A., Clements A.C. (2020). Mapping tuberculosis prevalence in Ethiopia: protocol for a geospatial meta-analysis. BMJ Open.

[24] Gething P.W., Casey D.C., Weiss D.J. (2016). Mapping plasmodium falciparum mortality in Africa between 1990 and 2015. N Engl J Med.

[25] Bhatt S., Weiss D., Cameron E. (2015). The effect of malaria control on Plasmodium falciparum in Africa between 2000 and 2015. Nature.

[26] Weiss D.J., Lucas T.C., Nguyen M. (2019). Mapping the global prevalence, incidence, and mortality of Plasmodium falciparum, 2000–17: a spatial and temporal modelling study. Lancet.

[27] Lessler J., Moore S.M., Luquero F.J. (2018). Mapping the burden of cholera in sub-Saharan Africa and implications for control: an analysis of data across geographical scales. Lancet.

[28] Chen Y., Ong J.H.Y., Rajarethinam J., Yap G., Ng L.C., Cook A.R. (2018). Neighbourhood level real-time forecasting of dengue cases in tropical urban Singapore. BMC Med.

[29] Karagiannis-Voules D.-A., Biedermann P., Ekpo U.F. (2015). Spatial and temporal distribution of soil-transmitted helminth infection in sub-Saharan Africa: a systematic review and geostatistical meta-analysis. Lancet Infect Dis.

[30] Chammartin F., Scholte R.G., Guimarães L.H., Tanner M., Utzinger J., Vounatsou P. (2013). Soil-transmitted helminth infection in South America: a systematic review and geostatistical meta-analysis. Lancet Infect Dis.

[31] Scholte R.G., Schur N., Bavia M.E. (2013).

[32] Tsheten T., Alene K.A., Restrepo A.C. (2024). Risk mapping and socio-ecological drivers of soil-transmitted helminth infections in the Philippines: a spatial modelling study. Lancet Reg Health West Pac.

[33] Gilmour B., Wangdi K., Restrepo A.C. (2024). Protocol for spatial prediction of soil transmitted helminth prevalence in the Western Pacific region using a meta-analytical approach. Syst Rev.

[34] Hodges J.S., Reich B.J. (2010). Adding spatially-correlated errors can mess up the fixed effect you love. Am Stat.

[35] Page M.J., McKenzie J.E., Bossuyt P.M. (2021). The PRISMA 2020 statement: an updated guideline for reporting systematic reviews. Int J Surg.

[36] World Health Organization Countries n.d.D. https://www.who.int/countries.

[37] Wells G.A.S.B., O’Connell D., Peterson J. (2014).

[38] University of Southampton WorldPop. n.d.. https://www.worldpop.org/.

[39] Weiss D., Nelson A., Vargas-Ruiz C. (2020). Global maps of travel time to healthcare facilities. Nat Med.

[40] Fick S.E., Hijmans R.J. (2017). WorldClim 2: new 1-km spatial resolution climate surfaces for global land areas. Int J Climatol.

[41] Farr T.G., Kobrick M. (2000). Shuttle radar Topography mission produces a wealth of data. Eos Trans AGU.

[42] International Soil Reference and Information Centre (ISRIC). World Soil Information. https://www.isric.org/.

[43] European Space Agency Earth online. n.d.. https://earth.esa.int/eogateway.

[44] Global Administrative Areas GADM maps and data. n.d.. https://gadm.org/.

[45] Candela E., Goizueta C., Sandon L., Muñoz-Antoli C., Periago M.V. (2023). The relationship between soil-transmitted helminth infections and environmental factors in Puerto Iguazú, Argentina: cross-sectional study. JMIR Public Health Surveill.

[46] Yaro C.A., Kogi E., Luka S.A. (2021). Edaphic and climatic factors influence on the distribution of soil transmitted helminths in Kogi East, Nigeria. Sci Rep.

[47] Rue H., Martino S., Chopin N. (2009). Approximate Bayesian inference for latent Gaussian models by using integrated nested Laplace approximations. J R Stat Soc Series B Stat Methodol.

[48] Lindgren F., Rue H., Lindström J. (2011). An explicit link between Gaussian fields and Gaussian Markov random fields: the stochastic partial differential equation approach. J R Stat Soc Series B Stat Methodol.

[49] Fuglstad G.-A., Simpson D., Lindgren F., Rue H. (2019). Constructing priors that penalize the complexity of Gaussian random fields. J Am Stat Assoc.

[50] Savioli L., Daumiere D., Savioli D. (2012).

[51] Agrawal R., Pattnaik S., Kshatri J.S. (2024). Prevalence and correlates of soil-transmitted helminths in schoolchildren aged 5 to 18 years in low-and middle-income countries: a systematic review and meta-analysis. Front Public Health.

[52] Requena-Mendez A., Buonfrate D., Bisoffi Z., Gutiérrez J.M. (2014). Advances in the diagnosis of human strongyloidiasis. Curr Trop Med Rep.

[53] Asundi A., Beliavsky A., Liu X.J. (2019). Prevalence of strongyloidiasis and schistosomiasis among migrants: a systematic review and meta-analysis. Lancet Global Health.

[54] Yang R., Xu M., Zhang L. (2025). Human strongyloides stercoralis infection. J Microbiol Immunol Infect.

[55] Gordon C.A., Kurscheid J., Jones M.K., Gray D.J., McManus D.P. (2017). Soil-transmitted helminths in tropical Australia and Asia. Trop Med Infect Dis.

[56] Salvador F., Trevino B., Sulleiro E. (2024). Epidemiological and clinical trends of imported strongyloidiasis in a referral international health unit, Barcelona, Spain: a 12-year period experience. Trav Med Infect Dis.

[57] Hasegawa M., Pilotte N., Kikuchi M. (2020). What does soil-transmitted helminth elimination look like? Results from a targeted molecular detection survey in Japan. Parasit Vectors.

[58] World Health Organization (2024).

[59] World Health Organization (2012).

[60] United Nations Development Programme (2024). Human development index (HDI). https://hdr.undp.org/data-center/human-development-index#/indicies/HDI.

[61] Raw C., Traub R.J., Zendejas-Heredia P.A., Stevenson M., Wiethoelter A. (2022). A systematic review and meta-analysis of human and zoonotic dog soil-transmitted helminth infections in Australian Indigenous communities. PLoS Negl Trop Dis.

[62] Vanalli C., Mari L., Casagrandi R., Gatto M., Cattadori I.M. (2024). Helminth ecological requirements shape the impact of climate change on the hazard of infection. Ecol Lett.

[63] Moncayo A.L., Lovato R., Cooper P.J. (2018). Soil-transmitted helminth infections and nutritional status in Ecuador: findings from a national survey and implications for control strategies. BMJ Open.

[64] Morales M.L., Lopez M., Ly P. (2019). Strongyloides stercoralis infection at different altitudes of the Cusco region in Peru. Am J Trop Med Hyg.

[65] Sumboh J.G., Agyenkwa-Mawuli K., Schwinger E. (2023). Investigating environmental determinants of soil-transmitted helminths transmission using GPS tracking and metagenomics technologies. medRxiv.

[66] Oyewole O.E., Simon-Oke I.A. (2022). Ecological risk factors of soil-transmitted helminths infections in Ifedore district, Southwest Nigeria. Bull Natl Res Cent.

[67] Tchuenté L.T. (2011). Control of soil-transmitted helminths in sub-Saharan Africa: diagnosis, drug efficacy concerns and challenges. Acta Trop.

[68] World Health Organization (2020).

[69] Bostoen K., Bilukha O.O., Fenn B. (2007).

[70] Weiss P.S., Michael E., Richards F.O. (2021). Simulating a transmission assessment survey: an evaluation of current methods used in determining the elimination of the neglected tropical disease, lymphatic filariasis. Int J Infect Dis.

[71] Kabore A., Biritwum N.-K., Downs P.W., Soares Magalhaes R.J., Zhang Y., Ottesen E.A. (2013). Predictive vs. empiric assessment of schistosomiasis: implications for treatment projections in Ghana. PLoS Negl Trop Dis.

