# Supplementary material for: Mapping the prevalence of soil-transmitted helminth infections in the Western Pacific Region: a spatial modelling study
Source: Lancet Reg Health West Pac. 2025 May 28;60:101581. doi: 10.1016/j.lanwpc.2025.101581 (PMC12417367; doi:10.1016/j.lanwpc.2025.101581)
Supplement: Supplementary File [file mmc1.pdf]

Mapping the prevalence of soil-transmitted helminth infections in the Western Pacific Region: a spatial modelling study: Supplementary Information

Contents

Supplementary Table 1: Methods 2
Supplementary Table 2: Variables included in the final analysis, including definitions and data sources. 3
Supplementary Table 3: PRISMA 2020 Checklist (1) 4
Supplementary Table 4: Characteristics of included studies 6
Supplementary Table 5 : QA assessment of STH studies based on modified Newcastle-Ottawa Quality Assessment Scale 14
Supplementary Table 6: Variance inflation factor (VIF) results for variables evaluated in the analysis. 23
Supplementary Table 7: Watanabe–Akaike information criterion results to select the best fitting model to predict STH infection in the WPR. 24
Supplementary Table 8: Variables associated with the best fitting model to predict the infection prevalence of each STH species 25
Supplementary Table 9: Pooled and predicted prevalence estimates at the country level for each species of STH infection in the WPR 26
Supplementary Figure 1: PRISMA shortlisting process (1) of the included studies 27
Supplementary Figure 2: Prediction standard deviation plots for each STH species. 29
Supplementary Figure 3 Predicted geographical distribution of STH infections for 1998-2011 and 2012-2021 across the WPR. 31
Supplementary Figure 4: Predicted geographical distribution of STH infections detailed at administrative levels across the WPR 33
Supplementary Figure 5: Model Diagnostics plots 34
Supplementary Figure 6 : Predicted geographical distribution of STH infections and model validation plots 38

Supplementary Table 1: Methods

Data sources for the outcome: The main sources of data were STH prevalence surveys published in peer-reviewed journals and grey literature.

Search strategy: The search strategy was defined following the Preferred Reporting Items for Systematic Review and Meta-Analysis (PRISMA) guidelines,(1) and a published protocol.(2) A comprehensive systematic search for epidemiological studies published in PubMed, Scopus, ProQuest, Embase and Web of Science between January 2000 and September 2023 was undertaken. The WHO regional classification system was used to define the 27 countries within the WPR.(3) We used relevant Medical Subject Headings (MeSH) and keywords for STH infections. Reference lists of included studies were checked for additional studies.
The search also included grey literature and regional databases, and Google Scholar was used to identify related articles using forward and backward citation searching. A summary of the search terms is detailed below:

Descriptor	Search Terms
STH search terms	soil transmitted helminth* OR STH OR *Ascaris* OR *Trichuris* OR *Nectator* OR *Ancylostoma* OR hookworm* OR Strongyloides stercoralis OR strongyloides fuelleborni kellyi OR roundworm* OR whipworm* OR threadworm* OR geohelmint* OR nematod*
^Δ^ Countries within the WPR	Australia OR Brunei OR Cambodia OR China OR Cook Islands OR Fiji OR Japan OR Kiribati OR Lao OR Laos OR Malaysia OR Marshall Islands OR Micronesia OR Mongolia OR Nauru OR New Zealand OR Niue OR Palau OR Papua New Guinea OR Philippines OR Republic of Korea OR Samoa OR Singapore OR Solomon Islands OR Tonga OR Tuvalu OR Vanuatu OR Vietnam

Δ The WHO Global Burden of Disease (GBD) regional classification system[4] was used to define the countries located within the WPR.

Study selection and eligibility criteria: All articles identified from the data base search were imported into Endnote X9 (Clarivate Analytics). After removing duplicates, the articles were exported to Rayyan QCRI. Two authors (BG and TT) independently reviewed titles and abstracts and any disagreements were resolved through discussion, and dialogue with a third author where required. The shortlisted full articles were assessed based on the following eligibility criteria.
The inclusion criteria for this review focused on studies related to human infection with specific species of STH, including *Ascaris lumbricoides* (roundworms), *Trichuris trichiura* (whipworms), *Necator americanus*, *Ancylostoma duodenale*, *Ancylostoma ceylanicum*, *Ancylostoma caninum*, and *Ancylostoma braziliense* (hookworms), as well as *Strongyloides stercoralis* and *Strongyloides fuelleborni* (threadworms). The studies had to utilize random sampling techniques and provide sufficient data for calculating STH prevalence. Additionally, the studies needed to be conducted in countries within the WPR as defined by the WHO regional classification system(3). For studies that included both pre- and post-intervention surveys, only pre-intervention baseline data were extracted. Where there were duplicate surveys for a given location, the study with the most recent or comprehensive data set was included within the analysis.
Studies were excluded if they were case studies or case series involving fewer than ten participants. Articles not published in English, conference abstracts, posters, and scientific correspondence were also excluded, along with literature at the country level. Surveys not representing the general population or pre-school-aged and school-aged children (PSAC/SAC), as well as those involving transient populations such as recent refugee arrivals that did not represent the local geography, were not included. To ensure the inclusion of recent data, studies published pre-2000 or those with survey collection dates pre-1998 were excluded- a data collection date was also selected to mitigate long publication lead times.

Data extraction: Two authors (BG and TT) independently extracted data from the included studies into a Microsoft Excel (version 2016) spreadsheet. From each eligible study data were extracted on characteristics of the study (first author; year of publication; year of data collection; study location including administrative level geographic location; study site (e.g., school, community); characteristics of participants (population group such as child and adult, ethnicity); STH species; diagnostic method; and outcome of interest including number of participants screened and number of participants STH positive.

Quality assessment: The methodological quality of the included studies was evaluated using a modified version of the Newcastle-Ottawa Scale(NOS).(5) The NOS judges the quality of observational studies with three basic perspectives: selection of study groups (4 points), comparability of groups (2 points), and ascertainment of the outcome of interest (3 points). The overall score ranges from zero to nine, with low (0–4), medium (5–7), and high (8–9) quality groupings.

Meta-analysis: A separate meta-analysis was performed using a random-effects model to estimate the pooled prevalence of each STH species at national levels. We calculated the summary effect size (ES) estimates and 95% confidence intervals (95% CI). Model parameters were estimated using the Restricted Maximum Likelihood (REML) method. Heterogeneity between studies was assessed with the Chi-squared test and the I^2 statistic. To account for heterogeneity and outliers, subgroup analyses were conducted based on study characteristics such as population type (children, community, ethnic minority) and survey year. The survey years were categorized into 1998-2011 and 2012-2021 to examine changes in NTD prevalence before and after the launch of the NTD Roadmap 2012(6), which aimed to accelerate NTD control efforts. Predicted prevalence maps for each STH species at both time periods were produced. Pooled period prevalence for each period was calculated using the total population sampled across all included surveys as the denominator Sensitivity analysis using the trim-and-fill method was employed to adjust for publication bias.

Supplementary Table 2: Variables included in the final analysis, including definitions and data sources.

Variables	Indicators	Definition	Source
Climatic variables	Average temperature (°C)	Annual average mean temperature (°C)	WorldClime(7)
	Precipitation (mm)	Annual mean rainfall (mm)	WorldClime(7)
	Solar radiation (kJ m-2 day-1)	Annual mean solar radiation	WorldClime(7)
Ecological variables	Altitude	Elevation of the earth land surface in km (8)	Shuttle Radar Topography Mission (SRTM) (9)
	Distance to the nearest health facility (minutes)	Walking travel times in minutes to the nearest health facility	Malaria Atlas Project (MAP)(10)
	Distance to a water body (km)	Distance to the nearest inland water body in km	WorldPop(11)
	Population density (person per square km)	Number of people per square kilometre	WorldPop(11)
Soil characteristics	Bulk density of the fine earth fraction (cg/cm3)	Bulk density of the fine earth fraction in kg dm-3	Global gridded soil data(12)
	The volume fraction of coarse fragments (> 2 mm; %)	The volume fraction of coarse fragments (> 2 mm) in percentage	Global gridded soil data(12)
	Total nitrogen (cg/kg1)	Total nitrogen in cg kg-1	Global gridded soil data(12)
	Organic carbon density (hg/m3)	Organic carbon density in hg/m-3	Global gridded soil data(12)
	Soil pH (measured in water)	Soil pH as measured in water	Global gridded soil data(12)
	Sand (> 0.05 mm) in fine earth (%)	Sand (> 0.05 mm) in fine earth in percentage	Global gridded soil data(12)
	Silt (0.002-0.05 mm) in fine earth (%)	Silt (0.002-0.05 mm) in fine earth in percentage	Global gridded soil data(12)
	Soil organic carbon in fine earth (g/kg)	Soil organic carbon in fine earth in g kg-1	Global gridded soil data(12)
Land cover variables	Trees	Geographic area dominated by trees with a cover of 10% or more	Worldwide land cover data(13)
	Grassland	Geographic area dominated by natural herbaceous plants (grasslands, prairies, steppes, savannahs, pastures) with a cover of 10% or more.	Worldwide land cover data(13)
	Cropland	Land covered with annual cropland that is sowed/planted and harvestable at least once within the 12 months after the sowing or planting date	Worldwide land cover data(13)
	Built	Land covered by buildings, roads and other man-made structures such as railroads	Worldwide land cover data(13)
	Bare	Lands with exposed soil, sand, or rocks and never has more than 10 % vegetated cover during any time of the year.	Worldwide land cover data(13)
	Wetland	Land dominated by natural herbaceous vegetation (cover of 10% or more) that is permanently or regularly flooded by fresh, brackish or salt water	Worldwide land cover data(13)
Polygon shape file		Administrative boundaries for each country at level o and 1	Global Administrative Areas (GADM)(14)

Supplementary Table 3: PRISMA 2020 Checklist (1)

Section and Topic	Item #	Checklist item	Location where item is reported
**TITLE**			
Title	1	Identify the report as a systematic review.	
**ABSTRACT**			
Abstract	2	See the PRISMA 2020 for Abstracts checklist.	
**INTRODUCTION**			
Rationale	3	Describe the rationale for the review in the context of existing knowledge.	P4 L158-160
Objectives	4	Provide an explicit statement of the objective(s) or question(s) the review addresses.	P5 L160-164
**METHODS**			
Eligibility criteria	5	Specify the inclusion and exclusion criteria for the review and how studies were grouped for the syntheses.	Supplementary Table 1
Information sources	6	Specify all databases, registers, websites, organisations, reference lists and other sources searched or consulted to identify studies. Specify the date when each source was last searched or consulted.	Supplementary Table 1
Search strategy	7	Present the full search strategies for all databases, registers and websites, including any filters and limits used.	Supplementary Table 1
Selection process	8	Specify the methods used to decide whether a study met the inclusion criteria of the review, including how many reviewers screened each record and each report retrieved, whether they worked independently, and if applicable, details of automation tools used in the process.	Supplementary Table 1
Data collection process	9	Specify the methods used to collect data from reports, including how many reviewers collected data from each report, whether they worked independently, any processes for obtaining or confirming data from study investigators, and if applicable, details of automation tools used in the process.	Supplementary Table 1
Data items	10a	List and define all outcomes for which data were sought. Specify whether all results that were compatible with each outcome domain in each study were sought (e.g. for all measures, time points, analyses), and if not, the methods used to decide which results to collect.	Supplementary Table 1
	10b	List and define all other variables for which data were sought (e.g. participant and intervention characteristics, funding sources). Describe any assumptions made about any missing or unclear information.	Supplementary Table 1
Study risk of bias assessment	11	Specify the methods used to assess risk of bias in the included studies, including details of the tool(s) used, how many reviewers assessed each study and whether they worked independently, and if applicable, details of automation tools used in the process.	Supplementary Table 1
Effect measures	12	Specify for each outcome the effect measure(s) (e.g. risk ratio, mean difference) used in the synthesis or presentation of results.	Supplementary Table 1
Synthesis methods	13a	Describe the processes used to decide which studies were eligible for each synthesis (e.g. tabulating the study intervention characteristics and comparing against the planned groups for each synthesis (item #5)).	
	13b	Describe any methods required to prepare the data for presentation or synthesis, such as handling of missing summary statistics, or data conversions.	
	13c	Describe any methods used to tabulate or visually display results of individual studies and syntheses.	
	13d	Describe any methods used to synthesize results and provide a rationale for the choice(s). If meta-analysis was performed, describe the model(s), method(s) to identify the presence and extent of statistical heterogeneity, and software package(s) used.	Supplementary Table 1
	13e	Describe any methods used to explore possible causes of heterogeneity among study results (e.g. subgroup analysis, meta-regression).	Supplementary Table 1

Section and Topic	Item #	Checklist item	Location where item is reported
	13f	Describe any sensitivity analyses conducted to assess robustness of the synthesized results.	Supplementary Table 1
Reporting bias assessment	14	Describe any methods used to assess risk of bias due to missing results in a synthesis (arising from reporting biases).	Supplementary Table 1
Certainty assessment	15	Describe any methods used to assess certainty (or confidence) in the body of evidence for an outcome.	Supplementary Table 1
**RESULTS**			
Study selection	16a	Describe the results of the search and selection process, from the number of records identified in the search to the number of studies included in the review, ideally using a flow diagram.	Supplementary Fig 1
	16b	Cite studies that might appear to meet the inclusion criteria, but which were excluded, and explain why they were excluded.	Supplementary Fig 1
Study characteristics	17	Cite each included study and present its characteristics.	Supplementary Table 4
Risk of bias in studies	18	Present assessments of risk of bias for each included study.	Supplementary Table 5
Results of individual studies	19	For all outcomes, present, for each study: (a) summary statistics for each group (where appropriate) and (b) an effect estimate and its precision (e.g. confidence/credible interval), ideally using structured tables or plots.	Manuscript Tables 1, 2 & 3
Results of syntheses	20a	For each synthesis, briefly summarise the characteristics and risk of bias among contributing studies.	
	20b	Present results of all statistical syntheses conducted. If meta-analysis was done, present for each the summary estimate and its precision (e.g. confidence/credible interval) and measures of statistical heterogeneity. If comparing groups, describe the direction of the effect.	Supplementary Table 9
	20c	Present results of all investigations of possible causes of heterogeneity among study results.	
	20d	Present results of all sensitivity analyses conducted to assess the robustness of the synthesized results.	
Reporting biases	21	Present assessments of risk of bias due to missing results (arising from reporting biases) for each synthesis assessed.	
Certainty of evidence	22	Present assessments of certainty (or confidence) in the body of evidence for each outcome assessed.	Manuscript Tables 1, 2 & 3
**DISCUSSION**			
Discussion	23a	Provide a general interpretation of the results in the context of other evidence.	P18 L411-413
	23b	Discuss any limitations of the evidence included in the review.	P20 L475-488
	23c	Discuss any limitations of the review processes used.	P20 L475-490
	23d	Discuss implications of the results for practice, policy, and future research.	P20 L491-503
**OTHER INFORMATION**			
Registration and protocol	24a	Provide registration information for the review, including register name and registration number, or state that the review was not registered.	
	24b	Indicate where the review protocol can be accessed, or state that a protocol was not prepared.	P5 L172

Section and Topic	Item #	Checklist item	Location where item is reported
	24c	Describe and explain any amendments to information provided at registration or in the protocol.	
Support	25	Describe sources of financial or non-financial support for the review, and the role of the funders or sponsors in the review.	P2 L76<br>P20 L506-510
Competing interests	26	Declare any competing interests of review authors.	P20 L512
Availability of data, code and other materials	27	Report which of the following are publicly available and where they can be found: template data collection forms; data extracted from included studies; data used for all analyses; analytic code; any other materials used in the review.	P21 L521-524

Supplementary Table 4: Characteristics of included studies

Study #	First Author + Year of Publication	Country of Study	Survey Population	STH species	Spatial resolution
**1**	Abdulla, 2002	Malaysia	Children	*A.lumbricoides*, *T.trichiura*, *S.stercoralis*,	*District centroid*
**2**	Addun, 2021	Philippines	Children	*A.lumbricoides*, *T.trichiura*	*Community level*
**3**	Adli, 2019	Malaysia	Aboriginal/ethnic minority	Hookworm	*Community level*
**4**	Adli, 2020	Malaysia	Aboriginal/ethnic minority	*A.lumbricoides*, *T.trichiura*, Hookworm	*Community level*
**5**	Ahmad, 2013	Malaysia	Aboriginal/ethnic minority	*A.lumbricoides*, *T.trichiura*, Hookworm, *S.stercoralis*	*Community level*
**6**	Ahmed, 2012	Malaysia	Aboriginal/ethnic minority	*A.lumbricoides*, *T.trichiura*, Hookworm	*Community level*
**7**	Aini, 2007	Malaysia	Aboriginal/ethnic minority	*A.lumbricoides*, *T.trichiura*, Hookworm	*Community level*
**8**	Al-Delaimy, 2014	Malaysia	Aboriginal/ethnic minority	*A.lumbricoides*, *T.trichiura*, Hookworm	*Community level*
**9**	Al-Mekhlafi, 2005	Malaysia	Aboriginal/ethnic minority	*A.lumbricoides*, *T.trichiura*, Hookworm	*District centroid*
**10**	Al-Mekhlafi, 2006	Malaysia	Aboriginal/ethnic minority	*A.lumbricoides*, *T.trichiura*, Hookworm	*District centroid*
**11**	Al-Mekhlafi, 2007	Malaysia	Aboriginal/ethnic minority	*A.lumbricoides*, *T.trichiura*, Hookworm	*Community level*
**12**	Al-Mekhlafi, 2008A	Malaysia	Aboriginal/ethnic minority	*A.lumbricoides*, *T.trichiura*, Hookworm, STH	*Community level*
**13**	Al-Mekhlafi, 2008B	Malaysia	Aboriginal/ethnic minority	*A.lumbricoides*, *T.trichiura*, Hookworm	*Community level*
**14**	Al-Mekhlafi, 2019	Malaysia	Aboriginal/ethnic minority	*S.stercoralis*	*District centroid*
**15**	Aminuddin, 2020	Malaysia	Aboriginal/ethnic minority	*A.lumbricoides*, *T.trichiura*, Hookworm	*Community level*

**16**	Anees, 2003	Malaysia	Children	*A.lumbricoides*, *T.trichiura*, Hookworm	*District centroid*
**17**	Angal, 2015	Malaysia	Community	*T.trichiura*, *S.stercoralis*	*District centroid*
**18**	Ash, 2017	Laos	Aboriginal/ethnic minority	*A.lumbricoides*, *T.trichiura*, Hookworm	*Community level*
**19**	Bahk, 2018	Korea	Children	*A.lumbricoides*, *T.trichiura*, Hookworm	*District centroid*
**20**	Baldo, 2004	Philippines	Children	*A.lumbricoides*, *T.trichiura*, Hookworm	*Community level*
**21**	Balen, 2011	China	Community	*A.lumbricoides*, *T.trichiura*, Hookworm, STH	*Community level*
**22**	Batbatan, 2012	Philippines	Children	*A.lumbricoides*, *T.trichiura*, Hookworm, *S.stercoralis*	*Community level*
**23**	Belizario, 2004	Philippines	Children	*A.lumbricoides*, *T.trichiura*, Hookworm	*Community level*
**24**	Belizario, 2009	Philippines	Children	*A.lumbricoides*, *T.trichiura*, Hookworm	*Community level*
**25**	Belizario, 2011	Philippines	Children	*A.lumbricoides*, *T.trichiura*, Hookworm	*Community level*
**26**	Belizario, 2014A	Philippines	Children	*A.lumbricoides*, *T.trichiura*, Hookworm	*Community level*
**27**	Belizario, 2014B	Philippines	Children	*A.lumbricoides*, *T.trichiura*, Hookworm	*Community level*
**28**	Belizario, 2015A	Philippines	Children	*A.lumbricoides*, *T.trichiura*, Hookworm	*Community level*
**29**	Belizario, 2015B	Philippines	Children	*A.lumbricoides*, *T.trichiura*, Hookworm	*Community level*
**30**	Belizario, 2016	Philippines	Children	*A.lumbricoides*, *T.trichiura*, Hookworm	*Community level*
**31**	Belizario, 2022	Philippines	Children	*A.lumbricoides*, *T.trichiura*	*Community level*
**32**	Bieri, 2013	China	Children	*A.lumbricoides*, *T.trichiura*	*Community level*
**33**	Bi-xian, 2022	China		*A.lumbricoides*, *T.trichiura*, Hookworm	*District centroid*
**34**	Bradbury, 2017	Solomon Isl	Community	Hookworm	*Community level*
**35**	Brandon-Mong, 2017	Malaysia	Aboriginal/ethnic minority	*A.lumbricoides*, *T.trichiura*, Hookworm	*Community level*
**36**	Bustos, 2021	Philippines	Children	*A.lumbricoides*, *T.trichiura*, Hookworm	*Community level*
**37**	Cao, 2023	China	Community	*A.lumbricoides*, *T.trichiura*, Hookworm	*District centroid*
**38**	Casey, 2013	Vietnam	Community	*A.lumbricoides*, *T.trichiura*, Hookworm	*Community level*
**39**	Chai, 2009	Laos	Community	*A.lumbricoides*, *T.trichiura*, Hookworm	*Community level*
**40**	Chai, 2015	Laos		*A.lumbricoides*, *T.trichiura*, Hookworm	*Community level*
**41**	Chang, 2020	Cambodia		Hookworm	*Community level*
**42**	Chard, 2019	Laos	Community	*A.lumbricoides*, *T.trichiura*, Hookworm, *S.stercoralis*	*Community level*
**43**	Chard, 2020	Laos	Children + Community	*A.lumbricoides*, *T.trichiura*, Hookworm, *S.stercoralis*	*Community level*
**44**	Chen, 2021	China		*A.lumbricoides*, *T.trichiura*, Hookworm	*District centroid*

**45**	Chen, 2022	China		*A.lumbricoides*, *T.trichiura*, Hookworm	*District centroid*
**46**	Chen, 2023	China	Community	*A.lumbricoides*, *T.trichiura*, Hookworm	*Community level*
**47**	Chhakda, 2006	Cambodia	Community	*A.lumbricoides*, *T.trichiura*, Hookworm, *S.stercoralis*	*Community level*
**48**	Chin, 2016	Malaysia	Aboriginal/ethnic minority	*A.lumbricoides*, *T.trichiura*, Hookworm	*Community level*
**49**	Chu, 2014	Tonga	Children	*A.lumbricoides*, *T.trichiura*, Hookworm	*District centroid*
**50**	Conlan, 2012	Laos	Community	*A.lumbricoides*, *T.trichiura*, Hookworm, *S.stercoralis*	*District centroid*
**51**	De Gier, 2015	Cambodia	Children	*A.lumbricoides*, *T.trichiura*, Hookworm	*Community level*
**52**	De Gier, 2016 A	Vietnam	Children	*A.lumbricoides*, *T.trichiura*, Hookworm	*Community level*
**53**	De Gier, 2016 B	Cambodia	Children	Hookworm	*Community level*
**54**	De Guia, 2019	Philippines	Aboriginal/ethnic minority	STH	*Community level*
**55**	Diep, 2017	Vietnam	Children + Community	*S.stercoralis*	*District centroid*
**56**	Ellis, 2007	China		*A.lumbricoides*, *T.trichiura*, Hookworm	*Community level*
**57**	Elyana, 2016	Malaysia	Aboriginal/ethnic minority + Children	*A.lumbricoides*, *T.trichiura*, Hookworm	*District centroid*
**58**	Eom, 2014	Laos		*A.lumbricoides*, *T.trichiura*, Hookworm	*District centroid*
**59**	Erlanger, 2008	Laos	Community	*A.lumbricoides*, *T.trichiura*, Hookworm, *S.stercoralis*	*District centroid*
**60**	Feng, 2020	China		*A.lumbricoides*, *T.trichiura*, Hookworm	*Community level*
**61**	Forrer, 2015	Laos	Community	Hookworm	*Community level*
**62**	Forrer, 2016	Cambodia	Community	*S.stercoralis*	*Community level*
**63**	Forrer, 2017	Cambodia	Community	*T.trichiura*, Hookworm, *S.stercoralis*	*Community level*
**64**	Forrer, 2018	Cambodia	Community	Hookworm, *S.stercoralis*	*Community level*
**65**	Forrer, 2018 A	Cambodia	Community	*S.stercoralis*	*Community level*
**66**	Forrer, 2018B	Cambodia	Community	Hookworm, *S.stercoralis*	*Community level*
**67**	Forrer, 2019	Cambodia	Community	*S.stercoralis*	*Community level*
**68**	Gandhi, 2001	China		*A.lumbricoides*, *T.trichiura*, Hookworm	*Community level*
**69**	Geik, 2015	Malaysia	Aboriginal/ethnic minority	*A.lumbricoides*, *T.trichiura*, Hookworm	*Community level*
**70**	Ghani, 2013	Malaysia	Aboriginal/ethnic minority	*A.lumbricoides*	*Community level*
**71**	Gordon, 2015	Philippines		*A.lumbricoides*, Hookworm	*District centroid*
**72**	Gou, 2019	China		*A.lumbricoides*, *T.trichiura*, Hookworm	*Community level*
**73**	Gyoten, 2010	Vietnam	Children	*A.lumbricoides*	*Community level*

**74**	Hanapian, 2014	Malaysia	Aboriginal/ethnic minority	*A.lumbricoides*, *T.trichiura*, Hookworm	*Community level*
**75**	Hanieh, 2021	Australia	Aboriginal/ethnic minority	*T.trichiura*	*District centroid*
**76**	Hartini, 2013	Malaysia	Aboriginal/ethnic minority	*A.lumbricoides*, *T.trichiura*, Hookworm	*Community level*
**77**	Hohmann, 2021	Laos	Children	*A.lumbricoides*, *T.trichiura*, Hookworm	*District centroid*
**78**	Holt, 2017	Australia	Aboriginal/ethnic minority	*T.trichiura*, Hookworm, *S.stercoralis*	*District centroid*
**79**	Hong-ju, 2020	China		*A.lumbricoides*	*District centroid*
**80**	Hsiao, 2014	Solomon Isl	Children	*A.lumbricoides*, *T.trichiura*, Hookworm, *S.stercoralis*	*Community level*
**81**	Huat, 2012	Malaysia	Children	*T.trichiura*	*Community level*
**82**	Hughes, 2004	Pacific Isl	Children	STH	*Community level*
**83**	Inpankaew, 2014	Cambodia	Community	Hookworm	*Community level*
**84**	Jeyaprakasam, 2019	Malaysia	Aboriginal/ethnic minority	*A.lumbricoides*, *T.trichiura*, Hookworm	*Community level*
**85**	Jin, 2021	Laos		*A.lumbricoides*, *T.trichiura*, Hookworm	*Community level*
**86**	Jin, 2022	China		STH	*Community level*
**87**	Kaewpitoon, 2015	Laos	Children	STH	*District centroid*
**88**	Kamel, 2001	Malaysia	Aboriginal/ethnic minority	*A.lumbricoides*, *T.trichiura*, Hookworm	*Community level*
**89**	Kamel, 2022	Malaysia	Aboriginal/ethnic minority	*A.lumbricoides*, *T.trichiura*, Hookworm	*Community level*
**90**	Kan, 2016	Malaysia	Aboriginal/ethnic minority	*A.lumbricoides*, *T.trichiura*, Hookworm	*Community level*
**91**	Kearns, 2017	Australia	Aboriginal/ethnic minority	*S.stercoralis*	*District centroid*
**92**	Khieu, 2013	Cambodia	Children	*A.lumbricoides*, *T.trichiura*, Hookworm, *S.stercoralis*	*Community level*
**93**	Khieu, 2014A	Cambodia	Children	*S.stercoralis*	*Community level*
**94**	Khieu, 2014B	Cambodia	Community	Hookworm, *S.stercoralis*	*Community level*
**95**	Khieu, 2014C	Cambodia	Community	*A.lumbricoides*, *T.trichiura*, Hookworm, *S.stercoralis*	*Community level*
**96**	Khir, 2020	Malaysia	Aboriginal/ethnic minority	*A.lumbricoides*, *T.trichiura*, Hookworm	*Community level*
**97**	Kim, 2003	Philippines		*A.lumbricoides*, *T.trichiura*, Hookworm	*District centroid*
**98**	Kim, 2016	Philippines	Children	*S.stercoralis*	*District centroid*
**99**	Kim, 2020	Fiji	Children	*A.lumbricoides*, *T.trichiura*, Hookworm	*Community level*
**100**	Kounnavong, 2011	Laos	Children	*A.lumbricoides*, *T.trichiura*, Hookworm	*District centroid*
**101**	Labana, 2021	Philippines	Children	*A.lumbricoides*, *T.trichiura*, Hookworm	*District centroid*
**102**	Lawangen, 2012	Philippines	Children	STH	*District centroid*

**103**	Laymanivong, 2014	Laos	Community	*A.lumbricoides*, *T.trichiura*, Hookworm	*Community level*
**104**	Laymanivong, 2016	Laos		*A.lumbricoides*, *T.trichiura*, Hookworm, *S.stercoralis*	*Community level*
**105**	Le, 2007	Vietnam	Children	*A.lumbricoides*, *T.trichiura*, Hookworm	*Community level*
**106**	Le, 2022	Solomon Isl		*A.lumbricoides*, *T.trichiura*, Hookworm, *S.stercoralis*	*District centroid*
**107**	Lee,(A) 2000	Philippines	Community	*A.lumbricoides*, *T.trichiura*, Hookworm	*Community level*
**108**	Lee, (B) 2000	China	Children	*A.lumbricoides*, *T.trichiura*, Hookworm	*Community level*
**109**	Lee, 2002	Cambodia	Children	*A.lumbricoides*, *T.trichiura*, Hookworm	*Community level*
**110**	Lee, 2014	Malaysia	Aboriginal/ethnic minority	*A.lumbricoides*, *T.trichiura*, Hookworm	*Community level*
**111**	Lee, 2017	China		*A.lumbricoides*, *T.trichiura*, Hookworm	*Community level*
**112**	Lee, 2021	Solomon Isl	Children	*A.lumbricoides*, *T.trichiura*, Hookworm, *S.stercoralis*	*Community level*
**113**	Liao, 2017A	Cambodia	Children	Hookworm, *S.stercoralis*	*District centroid*
**114**	Liao, 2017B	Marshall Is	Children	*T.trichiura*, Hookworm	*District centroid*
**115**	Lili, 2000	China	Aboriginal/ethnic minority + Community	*A.lumbricoides*, *T.trichiura*, Hookworm	*Community level*
**116**	Lim-Leroy, 2020	Malaysia		*A.lumbricoides*, *T.trichiura*, Hookworm	*Community level*
**117**	Lin, 2018	Fiji		*A.lumbricoides*, *T.trichiura*, Hookworm	*District centroid*
**118**	Linck, 2012	Laos		*A.lumbricoides*, *T.trichiura*, Hookworm, *S.stercoralis*	*Community level*
**119**	Liu, 2015	China	Children	*A.lumbricoides*, *T.trichiura*, Hookworm	*District centroid*
**120**	Liwang, 2017	Philippines		*A.lumbricoides*, *T.trichiura*, Hookworm	*District centroid*
**121**	Luo, 2023	China	Community	*A.lumbricoides*, *T.trichiura*, Hookworm	*District centroid*
**122**	Magalhaes, 2015	Philippines	Community	*A.lumbricoides*, *T.trichiura*, Hookworm	*Community level*
**123**	Maitong, 2017	Philippines	Children	*A.lumbricoides*, *T.trichiura*, Hookworm, *S.stercoralis*	*Community level*
**124**	Mationg, 2021	Philippines	Children	*A.lumbricoides*, *T.trichiura*, Hookworm	*Community level*
**125**	Mekhlafi, 2005	Malaysia	Aboriginal/ethnic minority	*A.lumbricoides*, *T.trichiura*, Hookworm	*Community level*
**126**	Mofid, 2010	China		*A.lumbricoides*, *T.trichiura*, Hookworm	*District centroid*
**127**	Mofid, 2011	China		*A.lumbricoides*, *T.trichiura*, Hookworm	*District centroid*
**128**	Mohd-Shaharuddin, 2018	Malaysia	Aboriginal/ethnic minority	*A.lumbricoides*, *T.trichiura*, Hookworm	*Community level*
**129**	Moktar, 2000	Malaysia	Aboriginal/ethnic minority	*A.lumbricoides*, *T.trichiura*, Hookworm	*Community level*
**130**	Murtaza, 2018	Malaysia	Aboriginal/ethnic minority	*A.lumbricoides*, *T.trichiura*	*District centroid*
**131**	Muslim, 2019	Malaysia	Aboriginal/ethnic minority	*A.lumbricoides*, *T.trichiura*, Hookworm, *S.stercoralis*	*Community level*

**132**	Nanthavong, 2017	Laos	Children	*A.lumbricoides*, *T.trichiura*, Hookworm	*District centroid*
**133**	Nasr, 2013	Malaysia	Aboriginal/ethnic minority	*A.lumbricoides*, *T.trichiura*, Hookworm	*Community level*
**134**	Nasr, 2020	Malaysia	Aboriginal/ethnic minority	*A.lumbricoides*, *T.trichiura*, Hookworm	*District centroid*
**135**	Ng, 2014	Philippines	Aboriginal/ethnic minority	*A.lumbricoides*, *T.trichiura*, Hookworm	*Community level*
**136**	Ngui, 2011	Malaysia		STH	*Community level*
**137**	Ngui, 2012	Malaysia		Hookworm	*Community level*
**138**	Ngui, 2015	Malaysia	Aboriginal/ethnic minority	*A.lumbricoides*, *T.trichiura*, Hookworm	*Community level*
**139**	Ngui, 2016	Malaysia	Aboriginal/ethnic minority	*S.stercoralis*	*Community level*
**140**	Nguyen, 2006	Vietnam	Community	*A.lumbricoides*, *T.trichiura*, Hookworm	*Community level*
**141**	Niamnuy, 2016	Laos	Community	Hookworm, *S.stercoralis*	*District centroid*
**142**	Nisha, 2015	Malaysia	Aboriginal/ethnic minority	*A.lumbricoides*, *T.trichiura*	*Community level*
**143**	Nisha, 2020	Malaysia	Aboriginal/ethnic minority	*A.lumbricoides*, *T.trichiura*	*Community level*
**144**	Noradilah, 2019	Malaysia	Aboriginal/ethnic minority	*A.lumbricoides*	*Community level*
**145**	Noradilah, 2022	Malaysia	Aboriginal/ethnic minority	*T.trichiura*	*Community level*
**146**	Olsen, 2006	Vietnam	Community	*A.lumbricoides*, *T.trichiura*, Hookworm	*District centroid*
**147**	Omran, 2020	Malaysia	Aboriginal/ethnic minority	*A.lumbricoides*, *T.trichiura*, Hookworm	*Community level*
**148**	Othman, 2020	Malaysia	Aboriginal/ethnic minority	*A.lumbricoides*, *T.trichiura*, Hookworm, *S.stercoralis*	*District level*
**149**	Park, 2004	Cambodia	Children	Hookworm	*Community level*
**150**	Pasricha, 2008	Vietnam	Community	Hookworm	*District centroid*
**151**	Peng, 2019	China		*A.lumbricoides*, *T.trichiura*, Hookworm	*District centroid*
**152**	Pham-Duc, 2013	Vietnam	Community	*A.lumbricoides*, *T.trichiura*, Hookworm	*Community level*
**153**	Phathammavong, 2007	Laos	Children	*A.lumbricoides*, *T.trichiura*, Hookworm, *S.stercoralis*	*Community level*
**154**	Phongluxa, 2013	Laos	Community	*A.lumbricoides*, *T.trichiura*, Hookworm	*District centroid*
**155**	Phuanukoonnon, 2013	PNG	Community	*A.lumbricoides*, *T.trichiura*, Hookworm, *S.stercoralis*	*District centroid*
**156**	Poise, 2020	Philippines		*A.lumbricoides*, *T.trichiura*, Hookworm	*Community level*
**157**	Rajoo, 2017	Malaysia	Aboriginal/ethnic minority	*A.lumbricoides*, *T.trichiura*, Hookworm	*Community level*
**158**	Ribas, 2017	Laos	Community	*A.lumbricoides*, *T.trichiura*, Hookworm, *S.stercoralis*	*Community level*
**159**	Rim, 2023	Laos	Children	*A.lumbricoides*, *T.trichiura*, Hookworm	*District centroid*
**160**	Ross, 2017A	Philippines		*A.lumbricoides*, *T.trichiura*, Hookworm	*District centroid*

**161**	Ross, 2017B	Philippines	Children	*A.lumbricoides*, *T.trichiura*, Hookworm	*District centroid*
**162**	Sagin, 2002	Malaysia	Aboriginal/ethnic minority	*A.lumbricoides*, *T.trichiura*, Hookworm	*Community level*
**163**	Sayasone, 2011	Laos	Aboriginal/ethnic minority	*A.lumbricoides*, *T.trichiura*, Hookworm, *S.stercoralis*	*Community level*
**164**	Sayasone, 2014	Laos	Children	*A.lumbricoides*, *T.trichiura*, Hookworm	*Community level*
**165**	Sayasone, 2015	Laos	Community	*A.lumbricoides*, *T.trichiura*, Hookworm	*Community level*
**166**	Sayasone, 2022	Laos	Community	*A.lumbricoides*, *T.trichiura*, Hookworm, *S.stercoralis*	*Community level*
**167**	Schar, 2013	Cambodia	Children	Hookworm, *S.stercoralis*	*Community level*
**168**	Schar, 2014	Cambodia	Community	*A.lumbricoides*, *T.trichiura*, Hookworm, *S.stercoralis*	*Community level*
**169**	Scott, 2022	PNG		*S.stercoralis*	*District centroid*
**170**	Senephansiri, 2017	Laos	Community	Hookworm, *S.stercoralis*	*Community level*
**171**	Shang, 2010	China	Children	*A.lumbricoides*, *T.trichiura*, Hookworm	*District centroid*
**172**	Shen, 2016	China		*A.lumbricoides*, *T.trichiura*, Hookworm	*District centroid*
**173**	Sinniah, 2012	Malaysia	Aboriginal/ethnic minority	*A.lumbricoides*, *T.trichiura*, Hookworm	*District centroid*
**174**	Sinuon, 2003	Cambodia	Children	*A.lumbricoides*, *T.trichiura*, Hookworm	*Community level*
**175**	Sithithaworn, 2006	Laos	Community	*A.lumbricoides*, *T.trichiura*, Hookworm, *S.stercoralis*	*District centroid*
**176**	Soriano, 2019	Philippines	Children	*A.lumbricoides*, *T.trichiura*, Hookworm	*Community level*
**177**	Speare, 2006	Tuvalu		*T.trichiura,* Hookworm	*District centroid*
**178**	Steinmann, 2007A	China	Aboriginal/ethnic minority	*A.lumbricoides*, *T.trichiura*, Hookworm, *S.stercoralis*	*Community level*
**179**	Steinmann, 2007B	China		A.lumbricoides, T.trichiura, Hookworm	*Community level*
**180**	Steinmann, 2008	China	Community	*A.lumbricoides*, *T.trichiura*, Hookworm, *S.stercoralis*	*Community level*
**181**	Steinmann, 2015	China	Community	*A.lumbricoides*, *T.trichiura*, Hookworm, *S.stercoralis*	*Community level*
**182**	Takano, 2009	Japan	Community	*S.stercoralis*	*Community level*
**183**	Tang, 2003	China	Community	*A.lumbricoides*, *T.trichiura*, Hookworm, *S.stercoralis*	*Community level*
**184**	Tang, 2019	Malaysia	Aboriginal/ethnic minority	*A.lumbricoides*, *T.trichiura*, Hookworm	*Community level*
**185**	Thanh, 2013	Vietnam	Community	Hookworm	*Community level*
**186**	Thomas, 2005	Fiji	Children	*A.lumbricoides*, *T.trichiura*, Hookworm	*Community level*
**187**	Tian, 2010	China	Community	*A.lumbricoides*, *T.trichiura*, Hookworm, *S.stercoralis*	*District centroid*
**188**	Tian-tian, 2022	China	Community	*A.lumbricoides*, *T.trichiura*, Hookworm	*District centroid*
**189**	Toma, 2000	Japan		*S.stercoralis*	*Community level*

**190**	Trang, 2007	Vietnam	Community	*A.lumbricoides*, *T.trichiura*	*Community level*
**191**	Trinos, 2019	Philippines	Children	*A.lumbricoides*, *T.trichiura*, Hookworm	*District centroid*
**192**	Tsheten, 2024	Philippines	Children	*A.lumbricoides*, *T.trichiura*, Hookworm	*Community level*
**193**	Tunbosun, 2017	Malaysia	Aboriginal/ethnic minority	*A.lumbricoides*, *T.trichiura*, Hookworm	*Community level*
**194**	Uga, 2005	Vietnam	Children	*A.lumbricoides*, *T.trichiura*, Hookworm	*Community level*
**195**	Verle, 2003	Vietnam		*A.lumbricoides*, *T.trichiura*, Hookworm	*District centroid*
**196**	Vonghachack, 2015A	Laos	Community	*A.lumbricoides*, *T.trichiura*, Hookworm, *S.stercoralis*	*Community level*
**197**	Vonghachack, 2015B	Laos		*A.lumbricoides*, *T.trichiura*, Hookworm, *S.stercoralis*	*Community level*
**198**	Vonghachack, 2017	Laos		*A.lumbricoides*, *T.trichiura*, Hookworm	*Community level*
**199**	Wang, 2012	China	Children	*A.lumbricoides*, *T.trichiura*, Hookworm	*Community level*
**200**	Wang, 2012A	China	Community	Hookworm	*Community level*
**201**	Wang, 2012B	China	Children	*A.lumbricoides*, *T.trichiura*, Hookworm	*Community level*
**202**	Wei-jun, 2022	China		*A.lumbricoides*, *T.trichiura*	*District centroid*
**203**	Wong, 2016	Malaysia	Aboriginal/ethnic minority	*A.lumbricoides*, *T.trichiura*, Hookworm	*District centroid*
**204**	Wong, 2021	Malaysia		*A.lumbricoides*, *T.trichiura*, Hookworm	*District centroid*
**205**	Xian-liang, 2021	China		*A.lumbricoides*, *T.trichiura*, Hookworm	*District centroid*
**206**	Xiao, 2015	China	Aboriginal/ethnic minority	*A.lumbricoides*, *T.trichiura*	*Community level*
**207**	Yajima, 2008	Vietnam		*A.lumbricoides*, *T.trichiura*, Hookworm	*Community level*
**208**	Yamamoto, 2000	Philippines	Children	*A.lumbricoides*, *T.trichiura*	*District centroid*
**209**	Yang, 2018	China	Children	*A.lumbricoides*, *T.trichiura*	*Community level*
**210**	Yap, 2012	China	Aboriginal/ethnic minority	*A.lumbricoides*, *T.trichiura*, Hookworm	*Community level*
**211**	Yap, 2013	China	Aboriginal/ethnic minority	*A.lumbricoides*, *T.trichiura*, Hookworm, *S.stercoralis*	*Community level*
**212**	Yong, 2011	Cambodia		*A.lumbricoides*, *T.trichiura*, Hookworm	*District centroid*
**213**	Yoshida, 2019	Laos	Children	*T.trichiura*, Hookworm	*District centroid*
**214**	Yosof, 2012	Malaysia	Aboriginal/ethnic minority	*T.trichiura*	*District centroid*
**215**	Yu, 2017	Philippines		*A.lumbricoides*, *T.trichiura*, Hookworm	*Community level*
**216**	Yuee, 2016	Malaysia	Aboriginal/ethnic minority	*A.lumbricoides*, *T.trichiura*, Hookworm	*Community level*
**217**	Yun-hong, 2022	Fiji		*A.lumbricoides*, *T.trichiura*, Hookworm	*Community level*
**218**	Zeng, 2019	China		A.lumbricoides, T.trichiura, Hookworm	*District centroid*

**219**	Zhang, 2019	China		*A.lumbricoides*	*District centroid*
**220**	Zhan-ying, 2020	China		*A.lumbricoides*	*District centroid*
**221**	Zhe, 2020	China		*A.lumbricoides*, *T.trichiura*, Hookworm	*District centroid*
**222**	Zhu, 2017	China		*A.lumbricoides*, *T.trichiura*, Hookworm	*District centroid*
**223**	Zhu, 2020	China		*A.lumbricoides*, *T.trichiura*, Hookworm	*District centroid*
**224**	Zhu, 2021	China		*A.lumbricoides*, *T.trichiura*, Hookworm	*District centroid*
**225**	Zhu, 2022	China		Hookworm	*District centroid*
**226**	Ziegelbauer, 2010	China	Aboriginal/ethnic minority + Children	*A.lumbricoides*, *T.trichiura*, Hookworm	*Community level*
**227**	Zulkifli, 2000	Malaysia	Children	*A.lumbricoides*, *T.trichiura*, Hookworm	*Community level*

Supplementary Table 5 : QA assessment of STH studies based on modified Newcastle-Ottawa Quality Assessment Scale

Study #	First Author, Year of publication	Study Population	Representativeness of the sample	Ascertainment of specimen collection methods	Sample size	Non-respondents	Impact of Bias (selection bias, measurement bias, participant reporting, confounders)	Assessment of the outcome (STH infection)	Statistical analysis	Total Score	QA Grade
**1**	Abdulla, 2002	1	2	1	0	0	0	1	0	5	medium
**2**	Addun, 2021	1	2	1	0	0	1	1	1	7	medium
**3**	Adli, 2019	1	2	1	0	0	0	1	0	5	medium
**4**	Adli, 2020	1	2	1	0	0	0	1	0	5	medium
**5**	Ahmad, 2013	1	2	1	1	0	1	1	1	8	high
**6**	Ahmed, 2012	1	2	1	0	1	1	1	1	8	high
**7**	Aini, 2007	1	2	1	0	1	0	1	1	7	medium
**8**	Al-Delaimy, 2014	1	2	1	1	1	1	1	1	9	high

**9**	Al-Mekhlafi, 2005	1	2	1	0	0	0	1	1	6	medium
**10**	Al-Mekhlafi, 2006	1	2	1	0	0	0	1	1	6	medium
**11**	Al-Mekhlafi, 2007	1	2	1	0	1	0	1	1	7	medium
**12**	Al-Mekhlafi, 2008A	1	2	1	1	1	0	1	1	8	high
**13**	Al-Mekhlafi, 2008B	1	2	1	0	0	0	1	1	6	medium
**14**	Al-Mekhlafi, 2019	1	2	1	1	1	1	1	1	9	high
**15**	Aminuddin, 2020	1	2	1	1	1	1	1	1	9	high
**16**	Anees, 2003	1	2	1	0	1	0	1	1	7	medium
**17**	Angal, 2015	1	2	1	1	0	1	1	1	8	high
**18**	Ash, 2017	1	2	1	0	1	1	1	1	8	high
**19**	Bahk, 2018	1	2	0	0	1	1	1	0	6	medium
**20**	Baldo, 2004	1	1	1	0	0	0	1	0	4	low
**21**	Balen, 2011	1	2	1	0	1	1	1	1	8	high
**22**	Batbatan, 2012	1	2	1	0	1	0	1	0	6	medium
**23**	Belizario, 2004	1	2	1	0	1	0	1	0	6	medium
**24**	Belizario, 2009	1	2	1	1	0	1	1	0	7	medium
**25**	Belizario, 2011	1	2	1	1	0	1	1	1	8	high
**26**	Belizario, 2014	1	1	1	1	0	0	1	1	6	medium
**27**	Belizario, 2014B	1	2	1	1	0	1	1	1	8	high
**28**	Belizario, 2015	1	2	1	0	1	1	1	1	8	high
**29**	Belizario, 2015	1	2	1	1	0	0	1	1	7	medium
**30**	Belizario, 2016	1	2	1	1	0	1	1	1	8	high
**31**	Belizario, 2022	1	2	1	1	0	1	1	1	8	high
**32**	Bieri, 2013	1	2	1	1	1	1	1	**1**	9	high
**33**	Bi-xian, 2022	1	1	1	0	0	0	1	1	5	medium
**34**	Bradbury, 2017	1	2	1	0	1	1	1	1	8	high
**35**	Brandon-Mong,2017	1	2	1	0	0	0	1	1	6	medium
**36**	Bustos, 2021	1	2	1	1	0	1	1	1	8	high
**37**	Cao, 2023	1	2	1	0	0	0	1	1	6	medium

**38**	Casey, 2013	1	1	1	1	1	1	1	1	8	high
**39**	Chai, 2009	1	0	1	0	0	1	1	1	5	medium
**40**	Chai, 2015	1	0	1	0	0	0	1	1	4	low
**41**	Chang, 2020	1	0	1	0	0	0	1	0	3	low
**42**	Chard, 2019	1	2	1	0	1	1	1	1	8	high
**43**	Chard, 2020	1	2	1	0	1	1	1	1	8	high
**44**	Chen, 2021	1	2	1	1	0	1	1	1	8	high
**45**	Chen, 2022	1	0	1	0	0	0	1	1	4	low
**46**	Chen, 2023	1	2	1	0	0	0	1	1	6	medium
**47**	Chhakda, 2006	1	1	1	0	0	0	1	1	5	medium
**48**	Chin, 2016	1	2	1	1	0	1	1	1	8	high
**49**	Chu, 2014	1	1	1	1	0	1	1	1	7	medium
**50**	Conlan, 2012	1	2	1	1	1	1	1	1	9	high
**51**	De Gier, 2015	1	0	1	0	0	1	1	1	5	medium
**52**	De Gier, 2016 A	1	2	1	0	1	1	1	1	8	high
**53**	De Gier, 2016 B	1	2	1	0	1	1	1	1	8	high
**54**	De Guia, 2019	1	0	1	1	1	0	1	0	5	medium
**55**	Diep, 2017	1	1	1	1	0	1	1	1	7	medium
**56**	Ellis, 2007	1	2	1	0	0	0	1	1	6	medium
**57**	Elyana, 2016	1	2	1	1	1	1	1	1	9	high
**58**	Eom, 2014	1	0	1	0	0	0	1	0	3	low
**59**	Erlanger, 2008	1	2	1	0	0	1	1	1	7	medium
**60**	Feng, 2020	1	2	1	0	0	0	1	1	6	medium
**61**	Forrer, 2015	1	2	1	0	1	1	1	1	8	high
**62**	Forrer, 2016	1	2	1	0	1	1	1	1	8	high
**63**	Forrer, 2017	1	2	1	0	1	1	1	1	8	high
**64**	Forrer, 2018	1	2	1	0	1	1	1	1	8	high
**65**	Forrer, 2018 A	1	2	1	0	1	1	1	1	8	high
**66**	Forrer, 2018B	1	2	1	0	1	1	1	1	8	high

**67**	Forrer, 2019	1	2	1	0	1	1	1	1	8	high
**68**	Gandhi, 2001	1	2	1	0	1	1	1	1	8	high
**69**	Geik, 2015	1	2	1	1	0	0	1	1	7	medium
**70**	Ghani, 2013	1	1	1	0	0	0	1	0	4	low
**71**	Gordon, 2015	1	1	1	0	0	1	1	1	6	medium
**72**	Gou, 2019	1	2	1	0	0	0	1	1	6	medium
**73**	Gyoten, 2010	1	0	0	0	0	0	1	1	3	low
**74**	Hanapian, 2014	1	2	1	0	0	0	1	1	6	medium
**75**	Hanieh, 2021	1	2	1	0	1	1	1	1	8	high
**76**	Hartini, 2013	1	2	1	0	1	0	1	1	7	medium
**77**	Hohmann, 2021	1	2	1	0	1	1	1	1	8	high
**78**	Holt, 2017	1	1	1	0	0	1	1	1	6	medium
**79**	Hong-ju, 2020	1	2	1	0	0	0	1	1	6	medium
**80**	Hsiao, 2014	1	2	0	0	1	0	1	0	5	medium
**81**	Huat, 2012	1	2	1	1	1	1	1	1	9	high
**82**	Hughes, 2004	1	1	1	0	1	0	1	1	6	medium
**83**	Inpankaew, 2014	1	2	1	0	0	0	1	1	6	medium
**84**	Jeyaprakasam, 2019	1	0	1	0	0	0	1	0	3	low
**85**	Jin, 2021	1	0	1	0	0	1	1	1	5	medium
**86**	Jin, 2022	1	0	1	0	0	0	1	1	4	low
**87**	Kaewpitoon, 2015	1	2	1	0	1	0	1	1	7	medium
**88**	Kamel, 2001	1	0	1	0	1	0	1	0	4	low
**89**	Kamel, 2022	1	0	1	0	0	0	1	0	3	low
**90**	Kan, 2016	1	0	1	0	1	0	1	0	4	low
**91**	Kearns, 2017	1	0	1	0	1	0	1	1	5	medium
**92**	Khieu, 2013	1	2	1	0	1	0	1	1	7	medium
**93**	Khieu, 2014A	1	2	1	0	1	1	1	1	8	high
**94**	Khieu, 2014B	1	2	1	0	1	1	1	1	8	high
**95**	Khieu, 2014C	1	2	1	0	1	1	1	1	8	high

**96**	Khir, 2020	1	2	1	0	1	1	1	1	8	high
**97**	Kim, 2003	1	0	1	0	0	0	1	0	3	low
**98**	Kim, 2016	1	1	1	0	0	1	1	0	5	medium
**99**	Kim, 2020	1	0	1	0	1	1	1	1	6	medium
**100**	Kounnavong, 2011	1	0	1	0	1	1	1	1	6	medium
**101**	Labana, 2021	1	0	1	1	1	0	1	1	6	medium
**102**	Lawangen, 2012	1	0	1	0	0	0	1	1	4	low
**103**	Laymanivong, 2014	1	2	1	1	0	0	1	0	6	medium
**104**	Laymanivong, 2016	1	2	1	0	0	0	1	1	6	medium
**105**	Le, 2007	1	0	1	0	0	0	1	1	4	low
**106**	Le, 2022	1	0	1	0	1	1	1	1	6	medium
**107**	Lee, 2000	1	0	1	0	0	0	1	0	3	low
**108**	Lee, 2000	1	0	1	0	1	0	1	1	5	medium
**109**	Lee, 2002	1	0	1	0	0	0	1	0	3	low
**110**	Lee, 2014	1	0	1	0	0	0	1	1	4	low
**111**	Lee, 2017	1	0	1	0	0	0	1	1	4	low
**112**	Lee, 2021	1	2	1	0	0	0	1	1	6	medium
**113**	Liao, 2017A	1	2	1	1	1	0	1	1	8	high
**114**	Liao, 2017B	1	0	1	1	1	0	1	1	6	medium
**115**	Lili, 2000	1	0	1	0	0	0	1	1	4	low
**116**	Lim-Leroy, 2020	1	0	1	1	1	0	1	1	6	medium
**117**	Lin, 2018	1	0	1	0	0	0	1	0	3	low
**118**	Linck, 2012	1	0	1	1	0	1	1	1	6	medium
**119**	Liu, 2015	1	2	1	0	0	1	1	1	7	medium
**120**	Liwang, 2017	1	0	1	1	1	0	1	1	6	medium
**121**	Luo, 2023	1	2	1	0	0	0	1	1	6	medium
**122**	Magalhaes, 2015	1	2	1	0	0	1	1	1	7	medium
**123**	Maitong, 2017	1	2	1	1	1	1	1	1	9	high
**124**	Mationg, 2021	1	0	1	1	0	1	1	1	6	medium

**125**	Mekhlafi, 2005	1	0	0	0	0	0	1	1	3	low
**126**	Mofid, 2010	1	2	1	0	0	0	1	0	5	medium
**127**	Mofid, 2011	1	0	1	0	0	0	1	1	4	low
**128**	Mohd-Shaharuddin, 2018	1	0	1	1	0	1	1	1	6	medium
**129**	Moktar, 2000	1	0	1	0	0	0	1	1	4	low
**130**	Murtaza, 2018	1	0	1	0	0	1	1	1	5	medium
**131**	Muslim, 2019	1	0	1	1	0	1	1	1	6	medium
**132**	Nanthavong, 2017	1	0	1	0	0	1	1	1	5	medium
**133**	Nasr, 2013	1	0	1	0	1	0	1	1	5	medium
**134**	Nasr, 2020	1	0	1	1	1	1	1	1	7	medium
**135**	Ng, 2014	1	0	1	0	1	1	1	1	6	medium
**136**	Ngui, 2011	1	0	1	0	0	0	1	1	4	low
**137**	Ngui, 2012	1	0	1	0	0	1	1	0	4	low
**138**	Ngui, 2015	1	2	1	1	0	1	1	1	8	high
**139**	Ngui, 2016	1	2	1	1	0	1	1	1	8	high
**140**	Nguyen, 2006	1	0	1	0	0	0	1	1	4	low
**141**	Niamnuy, 2016	1	2	1	0	0	0	1	1	6	medium
**142**	Nisha, 2015	1	0	1	0	0	1	1	1	5	medium
**143**	Nisha, 2020	1	0	1	1	0	0	1	1	5	medium
**144**	Noradilah, 2019	1	0	1	0	0	0	1	0	3	low
**145**	Noradilah, 2022	1	0	1	1	0	0	1	1	5	medium
**146**	Olsen, 2006	1	0	1	0	1	1	1	1	6	medium
**147**	Omran, 2020	1	0	1	0	0	0	1	0	3	low
**148**	Othman, 2020	1	2	1	1	0	1	1	1	8	high
**149**	Park, 2004	1	0	1	0	0	0	1	0	3	low
**150**	Pasricha, 2008	1	0	1	0	1	1	1	1	6	medium
**151**	Peng, 2019	1	2	0	0	0	0	1	1	5	medium
**152**	Pham-Duc, 2013	1	0	1	1	1	1	1	1	7	medium
**153**	Phathammavong, 2007	1	0	1	0	0	0	1	1	4	low

**154**	Phongluxa, 2013	1	0	1	0	1	1	1	1	6	medium
**155**	Phuanukoonnon, 2013	1	1	1	0	0	0	1	1	5	medium
**156**	Poise, 2020	1	0	1	0	0	0	1	1	4	low
**157**	Rajoo, 2017	1	0	1	1	0	1	1	1	6	medium
**158**	Ribas, 2017	1	2	1	0	0	0	1	1	6	medium
**159**	Rim, 2023	1	0	1	0	0	0	1	1	4	low
**160**	Ross, 2017A	1	0	1	0	0	0	1	1	4	low
**161**	Ross, 2017B	1	0	1	0	0	0	1	1	4	low
**162**	Sagin, 2002	1	2	1	0	0	0	1	0	5	medium
**163**	Sayasone, 2011	1	2	1	0	1	1	1	1	8	high
**164**	Sayasone, 2014	1	0	1	0	1	1	1	1	6	medium
**165**	Sayasone, 2015	1	2	1	0	1	1	1	1	8	high
**166**	Sayasone, 2022	1	0	1	0	1	1	1	1	6	medium
**167**	Schar, 2013	1	2	1	0	0	1	1	1	7	medium
**168**	Schar, 2014	1	2	1	0	0	0	1	1	6	medium
**169**	Scott, 2022	1	0	1	0	0	1	1	1	5	medium
**170**	Senephansiri, 2017	1	2	1	1	0	1	1	1	8	high
**171**	Shang, 2010	1	0	1	0	1	0	1	1	5	medium
**172**	Shen, 2016	1	0	1	0	0	0	1	0	3	low
**173**	Sinniah, 2012	1	0	1	0	0	0	1	0	3	low
**174**	Sinuon, 2003	1	0	1	0	0	0	1	0	3	low
**175**	Sithithaworn, 2006	1	2	1	0	1	0	1	1	7	medium
**176**	Soriano, 2019	1	2	1	0	1	1	1	1	8	high
**177**	Speare, 2006	1	0	1	0	1	0	1	1	5	medium
**178**	Steinmann, 2007A	1	2	1	0	1	1	1	1	8	high
**179**	Steinmann, 2007B	1	0	1	1	1	1	1	1	7	medium
**180**	Steinmann, 2008	1	2	1	0	1	1	1	1	8	high
**181**	Steinmann, 2015	1	2	1	1	0	1	1	1	8	high
**182**	Takano, 2009	1	2	1	0	0	0	1	1	6	medium

**183**	Tang, 2003	1	2	1	0	1	1	1	1	8	high
**184**	Tang, 2019	1	1	1	0	0	1	1	1	6	medium
**185**	Thanh, 2013	1	2	1	1	0	0	1	1	7	medium
**186**	Thomas, 2005	1	2	1	0	1	0	1	1	7	medium
**187**	Tian, 2010	1	2	1	0	1	1	1	1	8	high
**188**	Tian-tian, 2022	1	1	1	0	0	0	1	1	5	medium
**189**	Toma, 2000	1	0	1	0	0	0	1	0	3	low
**190**	Trang, 2007	1	2	1	1	1	1	1	1	9	high
**191**	Trinos, 2019	1	1	1	0	0	1	1	0	5	medium
**192**	Tsheten, 2024	1	2	1	0	0	1	1	1	7	medium
**193**	Tunbosun, 2017	1	2	1	1	1	0	1	1	8	high
**194**	Uga, 2005	1	0	1	0	1	0	1	1	5	medium
**195**	Verle, 2003	1	2	1	0	0	0	1	1	6	medium
**196**	Vonghachack, 2015A	1	2	1	0	1	0	1	1	7	medium
**197**	Vonghachack, 2015B	1	2	1	0	0	1	1	1	7	medium
**198**	Vonghachack, 2017	1	2	1	1	1	1	1	1	9	high
**199**	Wang, 2012	1	0	0	0	0	0	1	1	3	low
**200**	Wang, 2012A	1	0	1	0	0	0	1	1	4	low
**201**	Wang, 2012B	1	2	1	1	1	1	1	1	9	high
**202**	Wei-jun, 2022	1	1	1	0	0	0	1	1	5	medium
**203**	Wong, 2016	1	0	1	0	0	0	1	0	3	low
**204**	Wong, 2021	1	2	1	0	0	1	1	0	6	medium
**205**	Xian-liang, 2021	1	1	1	0	0	0	1	1	5	medium
**206**	Xiao, 2015	1	2	1	0	0	1	1	1	7	medium
**207**	Yajima, 2008	1	2	1	0	0	0	1	1	6	medium
**208**	Yamamoto, 2000	1	0	1	0	1	0	1	1	5	medium
**209**	Yang, 2018	1	2	1	0	1	1	1	1	8	high
**210**	Yap, 2012	1	2	1	0	1	1	1	1	8	high
**211**	Yap, 2013	1	2	1	1	1	0	1	1	8	high

**212**	Yong, 2011	1	0	0	0	0	1	1	1	4	low
**213**	Yoshida, 2019	1	1	1	1	0	1	1	1	7	medium
**214**	Yosof, 2012	1	0	1	0	0	0	1	1	4	low
**215**	Yu, 2017	1	1	1	0	0	0	1	1	5	medium
**216**	Yuee, 2016	1	2	1	1	0	1	1	1	8	high
**217**	Yun-hong, 2022	1	1	1	0	0	0	1	1	5	medium
**218**	Zeng, 2019	1	2	1	0	0	0	1	1	6	medium
**219**	Zhang, 2019	1	1	1	0	0	0	1	1	5	medium
**220**	Zhan-ying, 2020	1	1	1	0	0	0	1	1	5	medium
**221**	Zhe, 2020	1	1	1	0	0	0	1	1	5	medium
**222**	Zhu, 2017	1	0	1	0	0	0	1	1	4	low
**223**	Zhu, 2020 A	1	2	1	0	0	0	1	1	6	medium
**224**	Zhu, 2021	1	2	1	0	0	0	1	1	6	medium
**225**	Zhu, 2022	1	2	1	0	0	1	1	1	7	medium
**226**	Ziegelbauer, 2010	1	2	1	1	1	1	1	1	9	high
**227**	Zulkifli, 2000	1	2	1	0	1	1	1	1	8	high

On the basis of the modified Newcastle-Ottawa Quality Assessment Scale,(5) the average QA score across the studies included in the analysis was 6, therefore defining collective surveys to be of medium quality. It is noted however that 19 of the included studies had data extracted from the abstract and tables only as the remainder of the manuscript was not in English and so this QA score is potentially underestimated.

Supplementary Table 6: Variance inflation factor (VIF) results for variables evaluated in the analysis.

Variables	VIF				
	**All infections**	**Hookworm**	***Strongyloides***	***T. trichiura***	***A lumbricoides***
Climatic and other ecological variables					
Temperature	7.86	6.86	14.44	6.79	6.89
Precipitation	5.93	6.05	8.29	5.48	5.69
Altitude	4.17	2.64	10.9	2.83	2.91
Distance to health facility	1.51	1.54	2.84	1.55	1.52
Distance to water body	1.25	1.31	2.65	1.25	1.29
Solar radiation	2.86	3.25	7.05	3.11	2.68
Population density	1.84	1.89	7.94	1.87	1.82
Soil characteristics indicator variables					
Bulk density of the fine earth fraction	3.78	4.01	7.34	3.58	3.74
Volume fraction of coarse fragments	3.03	2.82	6.98	3.59	3.00
Nitrogen	3.53	3.89	19.19	3.46	3.47
Organic carbon density	6.58	7.58	18.69	6.59	7.08
PH	6.59	6.28	9.8	5.66	6.74
Sand	3.21	3.36	4.05	3.22	3.38
Silt	7.7	8.34	6.11	8.47	8.05
Organic carbon in fine earth	3.66	7.58	17.13	3.43	3.61
Land cover indicator variables					
Trees	24.9	22.9	134.89	26.9	13.99
Grass	6.21	4.26	17.34	4.47	3.04
Wetland	1.42	1.41	2.06	1.5	1.7
Crop	14.09	13.37	57.53	16.45	8.29
Buildings	12.47	12.33	72.19	13.87	7.65
Bare	3.48	3.11	5.83	3.31	2.83

Note: Variables with VIF value > 4 were excluded from the final model.

Supplementary Table 7: Watanabe–Akaike information criterion results to select the best fitting model to predict STH infection in the WPR.

Species	Models	WAIC
All infections	ALT	281077.73
	ALT + Dist_HF	278667.03
	ALT + Dist_HF + Dist_WB	301738.48
	ALT + Dist_HF + Dist_WB + Solar_R	280365.07
	ALT + Dist_HF + Dist_WB + Solar_R + Pop_Den	276105.47
	ALT + Dist_HF + Dist_WB + Solar_R + Pop_Den + bdod	284956.21
	ALT + Dist_HF + Dist_WB + Solar_R + Pop_Den + bdod + cfvo	286684.13
	ALT + Dist_HF + Dist_WB + Solar_R + Pop_Den + bdod + cfvo + Nitrogen	274756.42
	ALT + Dist_HF + Dist_WB + Solar_R + Pop_Den + bdod + cfvo + Nitrogen + Sand	273229.14
	ALT + Dist_HF + Dist_WB + Solar_R + Pop_Den + bdod + cfvo + Nitrogen + Sand + soc	272651.40
	ALT + Dist_HF + Dist_WB + Solar_R + Pop_Den + bdod + cfvo + Nitrogen + Sand + soc + Wet	274478.42
	ALT + Dist_HF + Dist_WB + Solar_R + Pop_Den + bdod + cfvo + Nitrogen + Sand + soc + Wet + Bare	266599.04
Hookworm	ALT	21672.80
	ALT + Dist_HF	21368.01
	ALT + Dist_HF + Dist_WB	21299.70
	ALT + Dist_HF + Dist_WB + Solar_R	21298.49
	ALT + Dist_HF + Dist_WB + Solar_R + Pop_Den	21003.32
	ALT + Dist_HF + Dist_WB + Solar_R + Pop_Den + bdod	20837.08
	ALT + Dist_HF + Dist_WB + Solar_R + Pop_Den + bdod + cfvo	19911.36
	ALT + Dist_HF + Dist_WB + Solar_R + Pop_Den + bdod + cfvo + Nitrogen	22809.72
	ALT + Dist_HF + Dist_WB + Solar_R + Pop_Den + bdod + cfvo + Nitrogen + Sand	28890.64
	ALT + Dist_HF + Dist_WB + Solar_R + Pop_Den + bdod + cfvo + Nitrogen + Sand + soc	33958.33
	ALT + Dist_HF + Dist_WB + Solar_R + Pop_Den + bdod + cfvo + Nitrogen + Sand + soc + Wet	39335.31
Strongyloids	Dist_HF	890.31
	Dist_HF + Dist_WB	840.32
	Dist_HF + Dist_WB + Pop_Den	822.17
	Dist_HF + Dist_WB + Pop_Den + bdod	810.03
	Dist_HF + Dist_WB + Pop_Den + bdod + cfvo	800.30
	Dist_HF + Dist_WB + Pop_Den + bdod + cfvo + Sand	771.12
	Dist_HF + Dist_WB + Pop_Den + bdod + cfvo + Sand + Silt	738.94
	Dist_HF + Dist_WB + Pop_Den + bdod + cfvo + Sand + Silt + Wet	738.62
	Dist_HF + Dist_WB + Pop_Den + bdod + cfvo + Sand + Silt + Wet + Bare	737.18
T.Trichiura	ALT	17230.67
	ALT + Dist_HF	17315.67
	ALT + Dist_HF + Dist_WB	16653.18
	ALT + Dist_HF + Dist_WB + Solar_R	16829.77
	ALT + Dist_HF + Dist_WB + Solar_R + Pop_Den	15375.81
	ALT + Dist_HF + Dist_WB + Solar_R + Pop_Den + bdod	15398.63
	ALT + Dist_HF + Dist_WB + Solar_R + Pop_Den + bdod + cfvo	16717.91
	ALT + Dist_HF + Dist_WB + Solar_R + Pop_Den + bdod + cfvo + Nitrogen	16619.81
	ALT + Dist_HF + Dist_WB + Solar_R + Pop_Den + bdod + cfvo + Nitrogen + Sand	17570.62
	ALT + Dist_HF + Dist_WB + Solar_R + Pop_Den + bdod + cfvo + Nitrogen + Sand + soc	16935.22
	ALT + Dist_HF + Dist_WB + Solar_R + Pop_Den + bdod + cfvo + Nitrogen + Sand + soc + Wet	16850.61
	ALT + Dist_HF + Dist_WB + Solar_R + Pop_Den + bdod + cfvo + Nitrogen + Sand + soc + Wet + Bare	16104.02
A.Lumbricoides	ALT	18999.85
	ALT + Dist_HF	19423.25
	ALT + Dist_HF + Dist_WB	18092.20
	ALT + Dist_HF + Dist_WB + Solar_R	18052.96
	ALT + Dist_HF + Dist_WB + Solar_R + Pop_Den	18491.49
	ALT + Dist_HF + Dist_WB + Solar_R + Pop_Den + bdod	18498.20
	ALT + Dist_HF + Dist_WB + Solar_R + Pop_Den + bdod + cfvo	18604.12
	ALT + Dist_HF + Dist_WB + Solar_R + Pop_Den + bdod + cfvo + Nitrogen	18463.61
	ALT + Dist_HF + Dist_WB + Solar_R + Pop_Den + bdod + cfvo + Nitrogen + Sand	18699.01
	ALT + Dist_HF + Dist_WB + Solar_R + Pop_Den + bdod + cfvo + Nitrogen + Sand + soc	18188.79
	ALT + Dist_HF + Dist_WB + Solar_R + Pop_Den + bdod + cfvo + Nitrogen + Sand + soc + Grass	18286.78
	ALT + Dist_HF + Dist_WB + Solar_R + Pop_Den + bdod + cfvo + Nitrogen + Sand + soc + Grass + Wet	17926.11
	ALT + Dist_HF + Dist_WB + Solar_R + Pop_Den + bdod + cfvo + Nitrogen + Sand + soc + Grass + Wet + Bare	19858.88

Note: The models shaded in blue colour have the smallest WAIC and variables included in these models were used to produce the final prediction maps

Key: ALT=altitude; Dist_HF=distance to health facility; Dist_WB=distance to water body; Solar_R=solar radiation; Pop_Den=population density; bdod=bulk density of the fine earth fraction; cfvo=volume fraction of coarse fragments; Nitrogen; Sand; soc=organic carbon in fine earth; Wet= wetlands; Bare

Supplementary Table 8: Variables associated with the best fitting model to predict the infection prevalence of each STH species

Variables associated with the best fitting model to predict infection prevalence					
	**All infections**	**Hookworm**	***Strongyloides***	***T. trichiura***	***A lumbricoides***
Climatic and other ecological variables					
Temperature	√				
Precipitation					
Altitude		√		√	√
Distance to health facility	√	√	√	√	√
Distance to water body	√	√	√	√	√
Solar radiation	√	√		√	√
Population density	√	√	√	√	√
Soil characteristics indicator variables					
Bulk density of the fine earth fraction	√	√	√		√
Volume fraction of coarse fragments	√	√	√		√
Nitrogen	√				√
Organic carbon density					
PH					
Sand	√		√		√
Silt			√		
Organic carbon in fine earth	√				√
Land cover indicator variables					
Trees					
Grass					√
Wetland	√		√		√
Crop					
Buildings					
Bare	√		√		

N.B. Models that determined the best estimates excluded VIF >4 for 'all infections', Hookworm, *T.trichiura* and *A.lumbricoides.* For *Strongyloides* the best fitting prediction was obtained when VIF >8 were excluded.

Supplementary Table 9: Pooled and predicted prevalence estimates at the country level for each species of STH infection in the WPR

Country	All infections		Hookworm		*Strongyloides*		*T. trichiura*		*A. lumbricoides*	
	**Pooled**	**Predicted**	**Pooled**	**Predicted**	**Pooled**	**Predicted**	**Pooled**	**Predicted**	**Pooled**	**Predicted**
Vietnam	44.5 (33.0, 56.7)	28.1 (5.6, 54.8)	26.8 (13.4, 46.3)	8.5 (1.3, 17.2)	13.4 (6.5, 25.7)	1.2 (0.4, 1.7)	26.6 (14.5, 43.8)	18.8 (7.2, 31.6)	25.8 (12.8, 45.0)	16.3 (7.2, 26.6)
Malaysia	41.5 (35.9, 47.4)	36 (18, 58.1)	11.3 (8.8, 14.5)	9.4 (3.0, 17.3)	9.7 (6.5, 14.4)	1.6 (0.7, 2.4)	44.7 (32.9, 57.1)	20.1 (11.0, 30.3)	27.1 (20.0, 35.6)	16.3 (7.6, 27.1)
Laos	32.6 (27.5, 38.1)	40.9 (32.7, 49.1)	28.8 (23.2, 35.1)	12.6 (8.7, 16.8)	6.7 (3.9, 11.4)	1.7 (1.0, 2.3)	12.1 (8.7, 16.6)	19.2 (14.9, 23.5)	11.3 (7.3, 17.2)	17.5 (13.3, 21.6)
Philippines	29.6 (27.2, 32.2)	38 (23.8, 54.5)	10.7 (9.0, 11.2)	7.8 (2.3, 14.6)	0.9 (0.3, 2.5)	1.3 (0.4, 1.9)	14.2 (12.5, 16.0)	20.5 (13.2, 28.5)	13.4 (12.0, 14.9)	15.7 (8.3, 23.7)
Solomon Isl.	28.4 (9.7, 59.4)	31.6 (6.9, 54.4)	14.2 (2.4, 53.1)	8.4 (0.06, 18.7)	5.7 (2.3, 13.3)	4.9 (4.8, 5.2)	8.2 (1.6, 32.8)	29 (26.3, 32.7)	1.8 (1.0, 26.4)	21.7 (21.1, 23.5)
Cambodia	24.4 (18.3, 31.7)	35.6 (29.2, 42.7)	13.1 (10.1, 16.9)	12 (8.7, 15.4)	23.9 (13.2, 39.6)	2.2 (1.3, 3.1)	2.1 (1.3, 3.3)	9.6 (7.1, 11.5)	16.1 (7.8, 30.2)	8.2 (5.1, 10.7)
PNG	8.6 (3.5, 19.6)	27.8 (0.9, 61.0)	NA	NA	8.7 (1.9, 31.9)	4.2 (3.5, 5.1)	NA	NA	NA	NA
Japan	6.4 (3.3, 12.0)	29.1 (0.4, 61.9)	NA	NA	6.4 (3.3, 12.0)	1.2 (0.2, 1.9)	NA	NA	NA	NA
China	4.3 (3.2, 5.8)	25.2 (0.7, 57.7)	3.2 (2.1, 4.9)	7.7 (0.1, 18.7)	0.04 (0, 2.1)	2.3 (1.5, 2.9)	6.5 (3.4, 12.1)	12.8 (0.6, 30.1)	6.9 (3.8, 12.1)	12.4 (0.2, 27.9)
Korea, Republic of	0.02 (0, 0.12)	25.5 (5.1, 58.1)	0	6.3 (0.01, 18.2)	NA	NA	0.05 (0.01, 0.4)	12.6 (0.06, 28.5)	0	10.4 (0.01, 24.5)

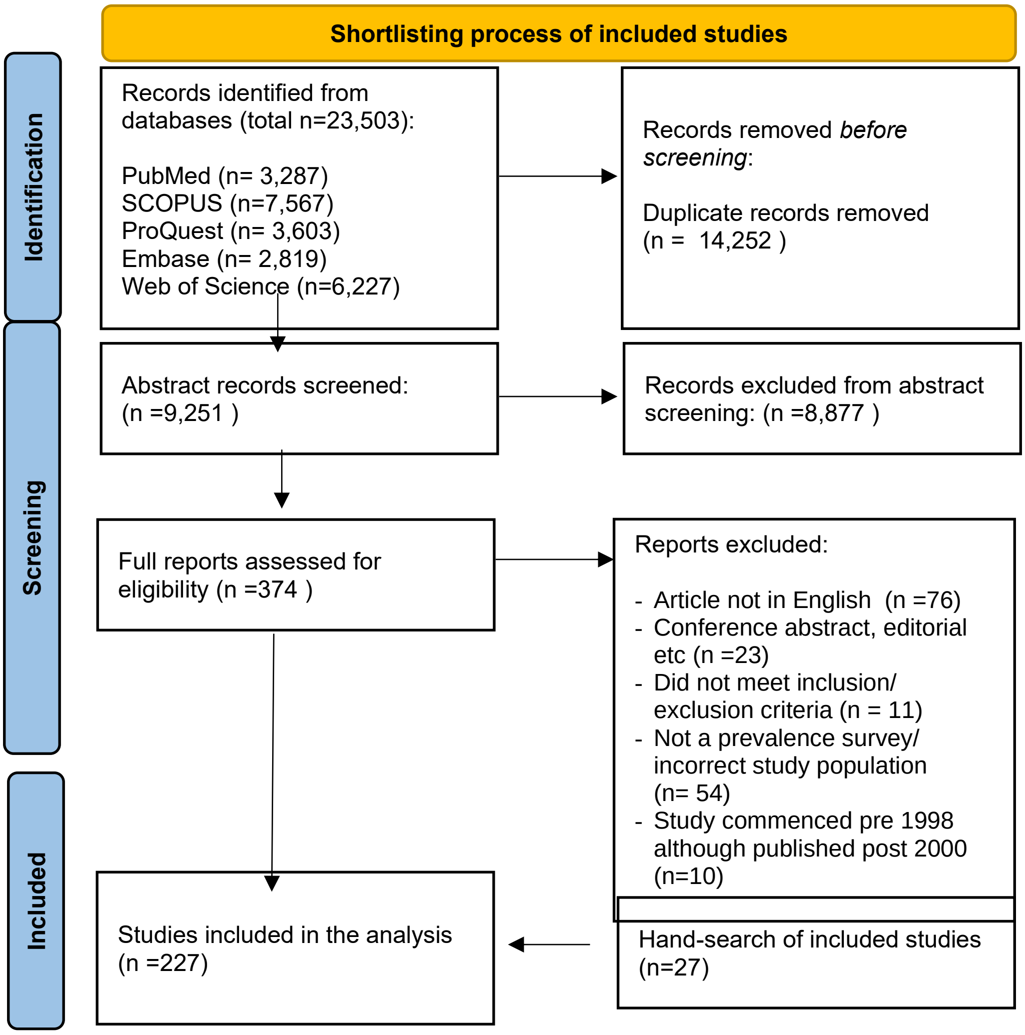

Supplementary Figure 1: PRISMA shortlisting process (1) of the included studies

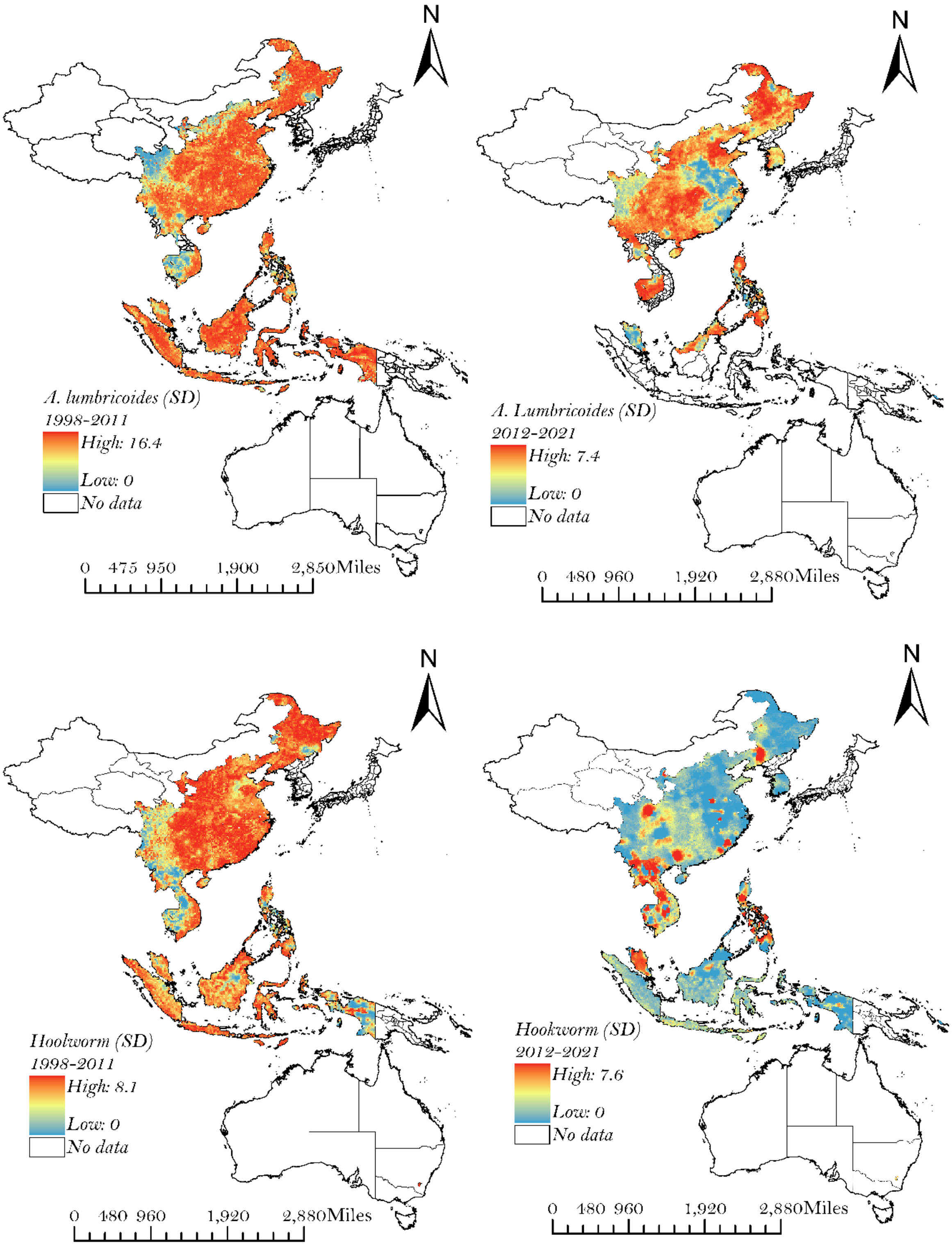
N
A. lumbricoides (SD)
1998-2011
High: 16.4
Low: 0
No data
0 475 950 1,900 2,850Miles
N
A. Lumbricoides (SD)
2012-2021
High: 7.4
Low: 0
No data
0 480 960 1,920 2,880Miles
N
Hoolworm (SD)
1998-2011
High: 8.1
Low: 0
No data
0 480 960 1,920 2,880Miles
N
Hookworm (SD)
2012-2021
High: 7.6
Low: 0
No data
0 480 960 1,920 2,880Miles

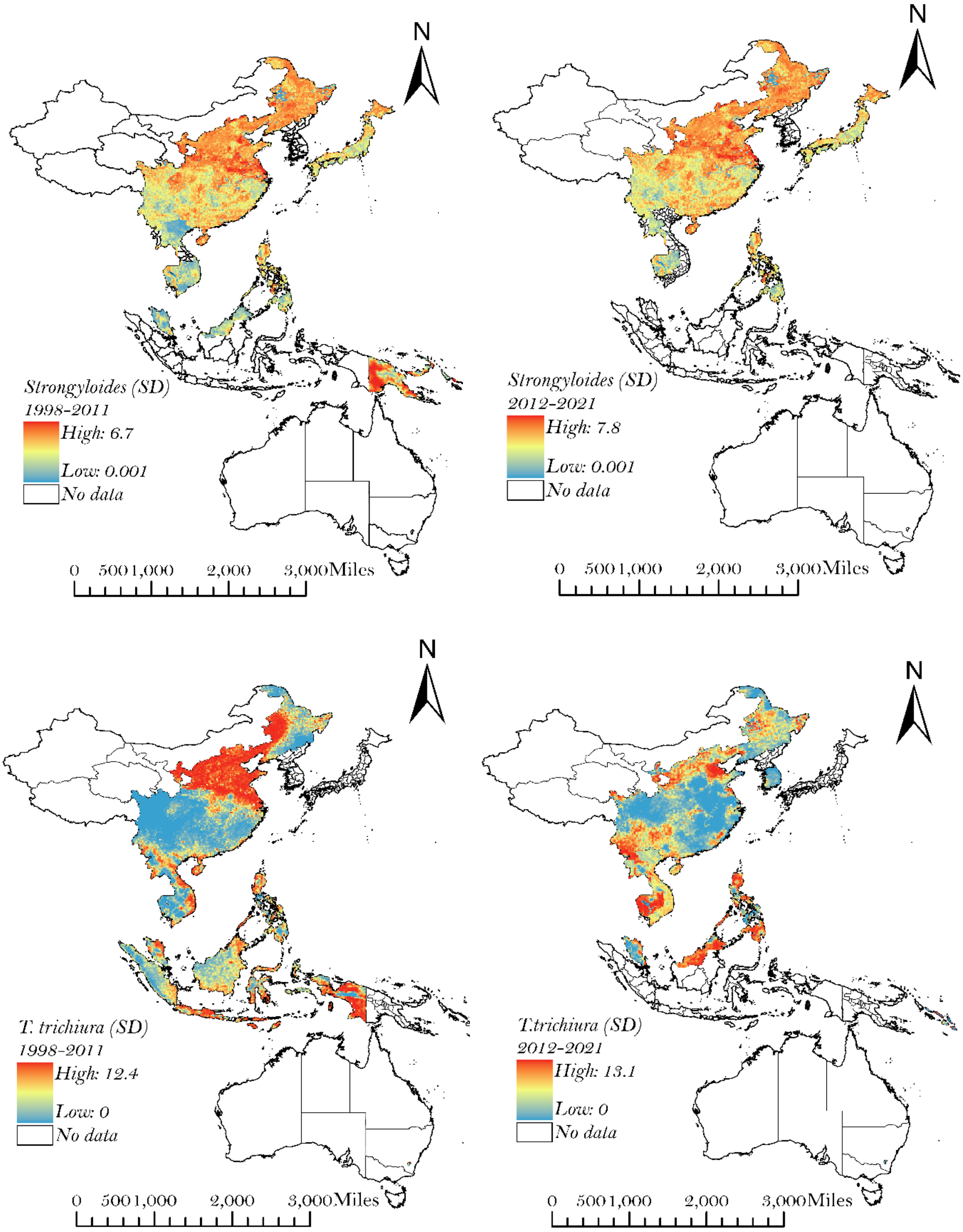

Supplementary Figure 2: Prediction standard deviation plots for each STH species.

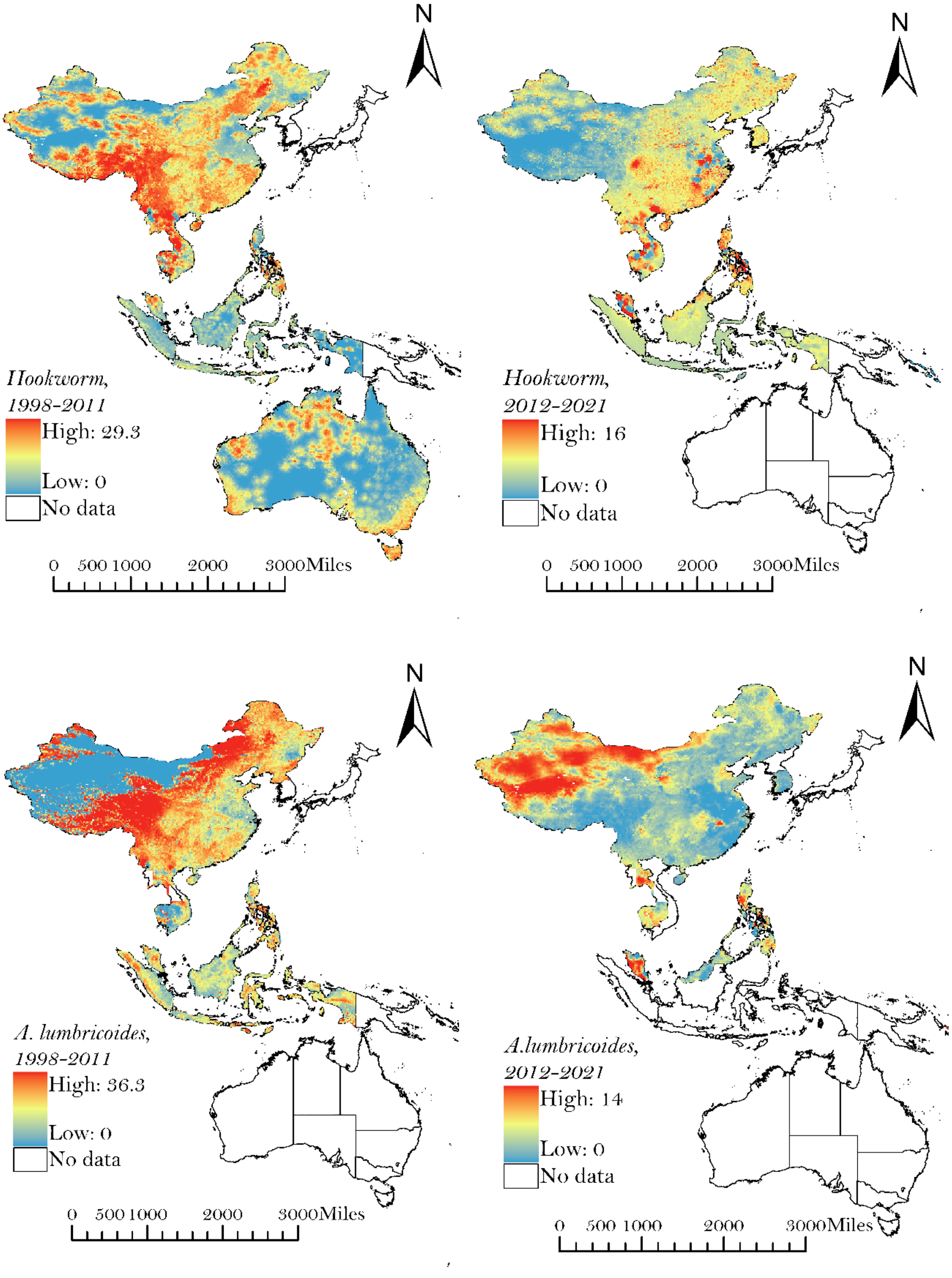
N
Hookworm,
1998-2011
High: 29.3
Low: 0
No data
0 500 1000 2000 3000Miles
N
Hookworm,
2012-2021
High: 16
Low: 0
No data
0 500 1000 2000 3000Miles
N
A. lumbricoides,
1998-2011
High: 36.3
Low: 0
No data
0 500 1000 2000 3000Miles
N
A.lumbricoides,
2012-2021
High: 14
Low: 0
No data
0 500 1000 2000 3000Miles

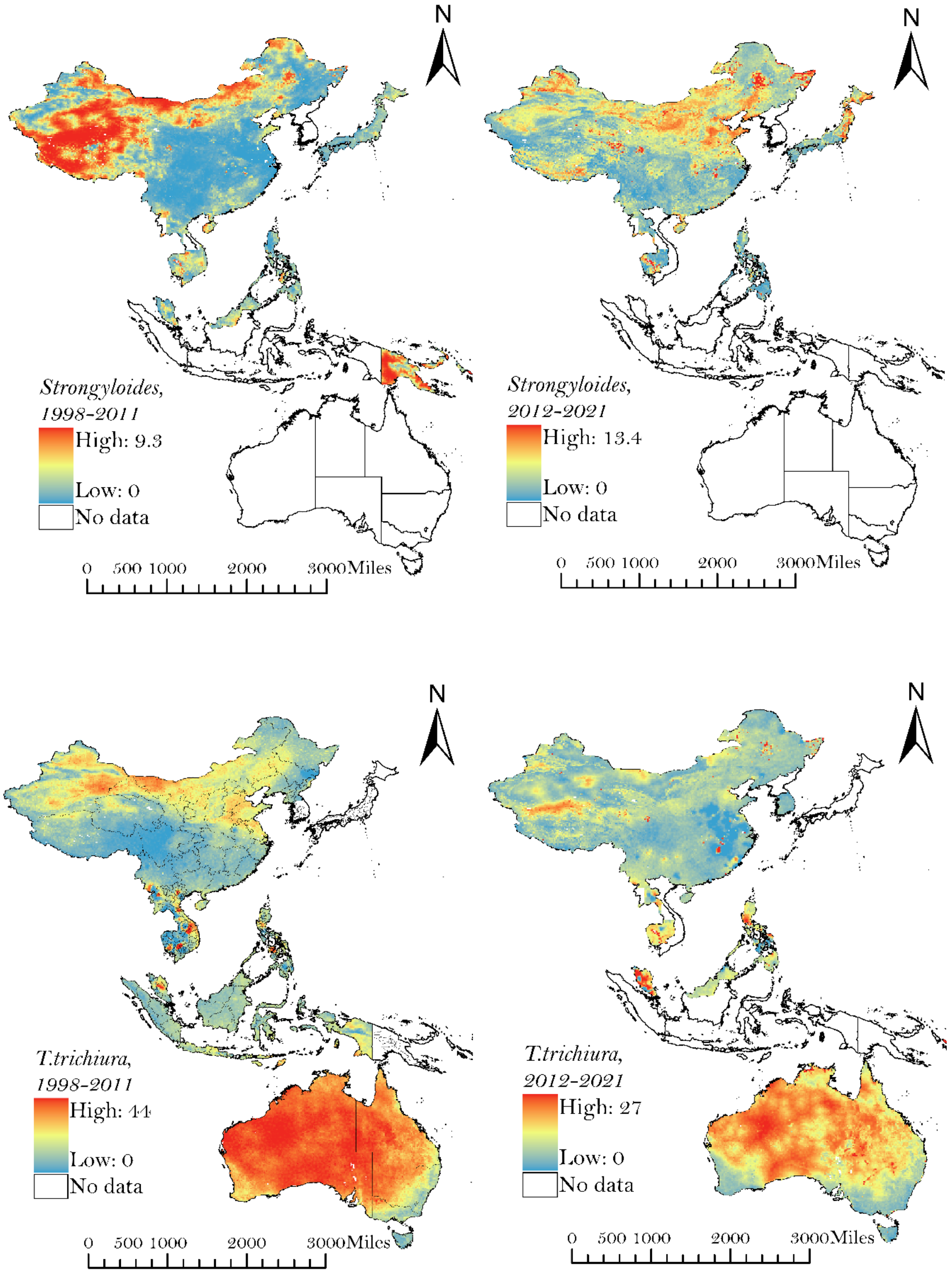

Supplementary Figure 3 Predicted geographical distribution of STH infections for 1998-2011 and 2012-2021 across the WPR.

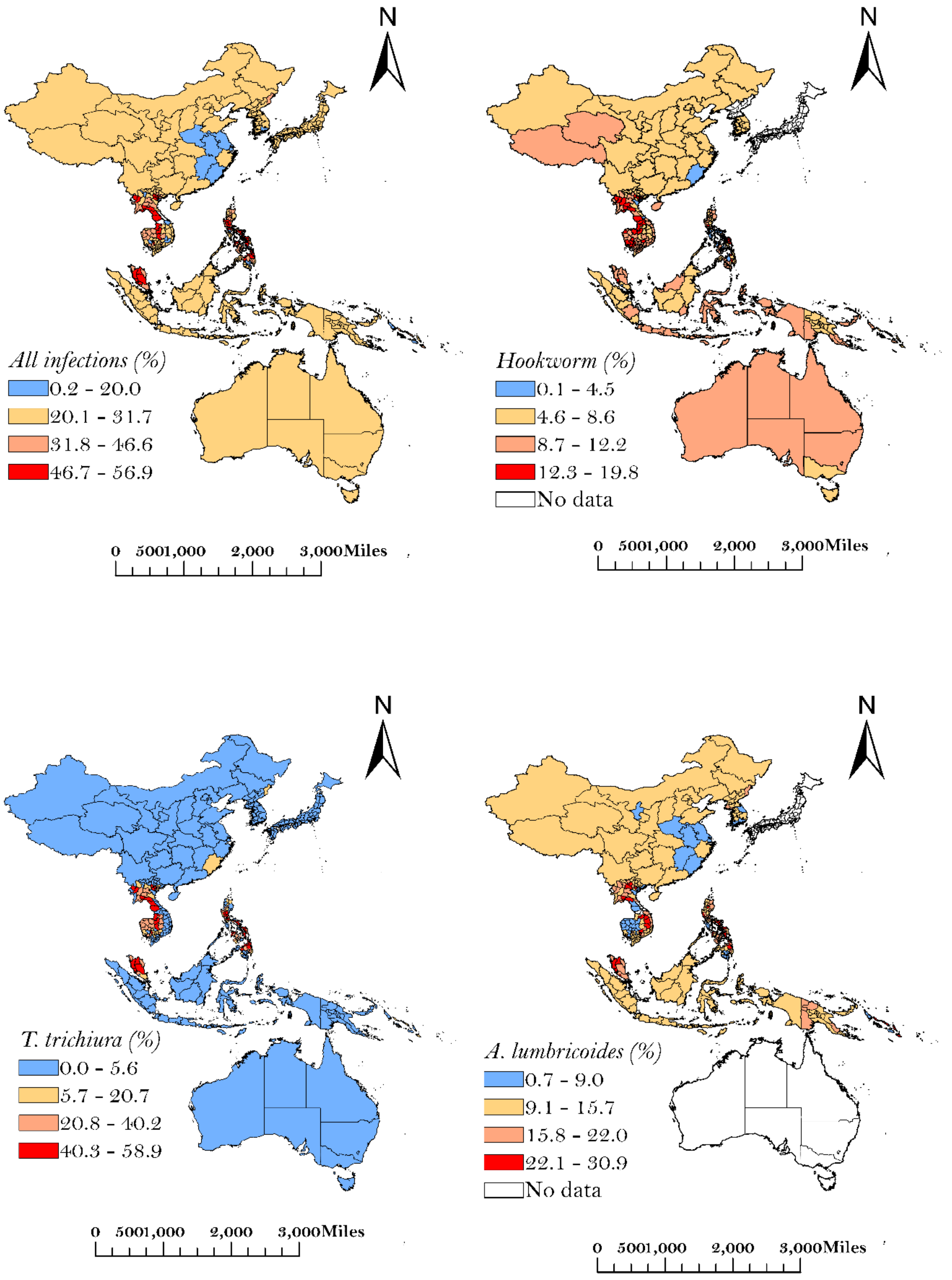
N
All infections (%)
0.2 - 20.0
20.1 - 31.7
31.8 - 46.6
46.7 - 56.9
0 500 1,000 2,000 3,000 Miles
N
Hookworm (%)
0.1 - 4.5
4.6 - 8.6
8.7 - 12.2
12.3 - 19.8
No data
0 500 1,000 2,000 3,000 Miles
N
T. trichiura (%)
0.0 - 5.6
5.7 - 20.7
20.8 - 40.2
40.3 - 58.9
0 500 1,000 2,000 3,000 Miles
N
A. lumbricoides (%)
0.7 - 9.0
9.1 - 15.7
15.8 - 22.0
22.1 - 30.9
No data
0 500 1,000 2,000 3,000 Miles

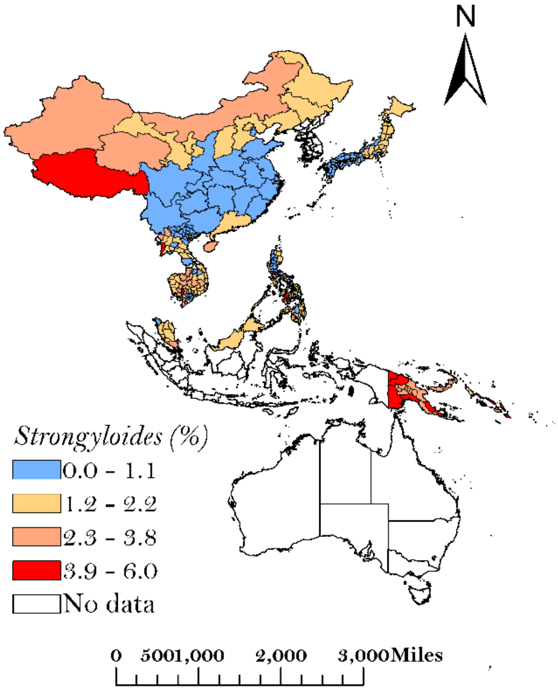

Supplementary Figure 4: Predicted geographical distribution of STH infections detailed at administrative levels across the WPR

Supplementary Figure 5: Model Diagnostics plots

We used two posterior predictive methods (CPO and PIT) to measure the predictive ability of our models' using parameters sampled from the posterior distribution. The CPO measures the fit of the model to an individual site: specifically, the CPO for a given site i equals $p(\mathbf{y}_i|\mathbf{y}_{(-i)})$: the probability of the data for that site ($\mathbf{y}_i$) conditional on the data ($\mathbf{y}_{(-i)}$) for all other sites. Its definition also makes CPO a cross-validation method because evaluation of a particular site is based on only data from other sites(15). In addition, PIT measures the probability of a new value to be lower than the actual observed value and for a well-calibrated model, the PIT values should be uniformly distributed.

A. lumbricoides

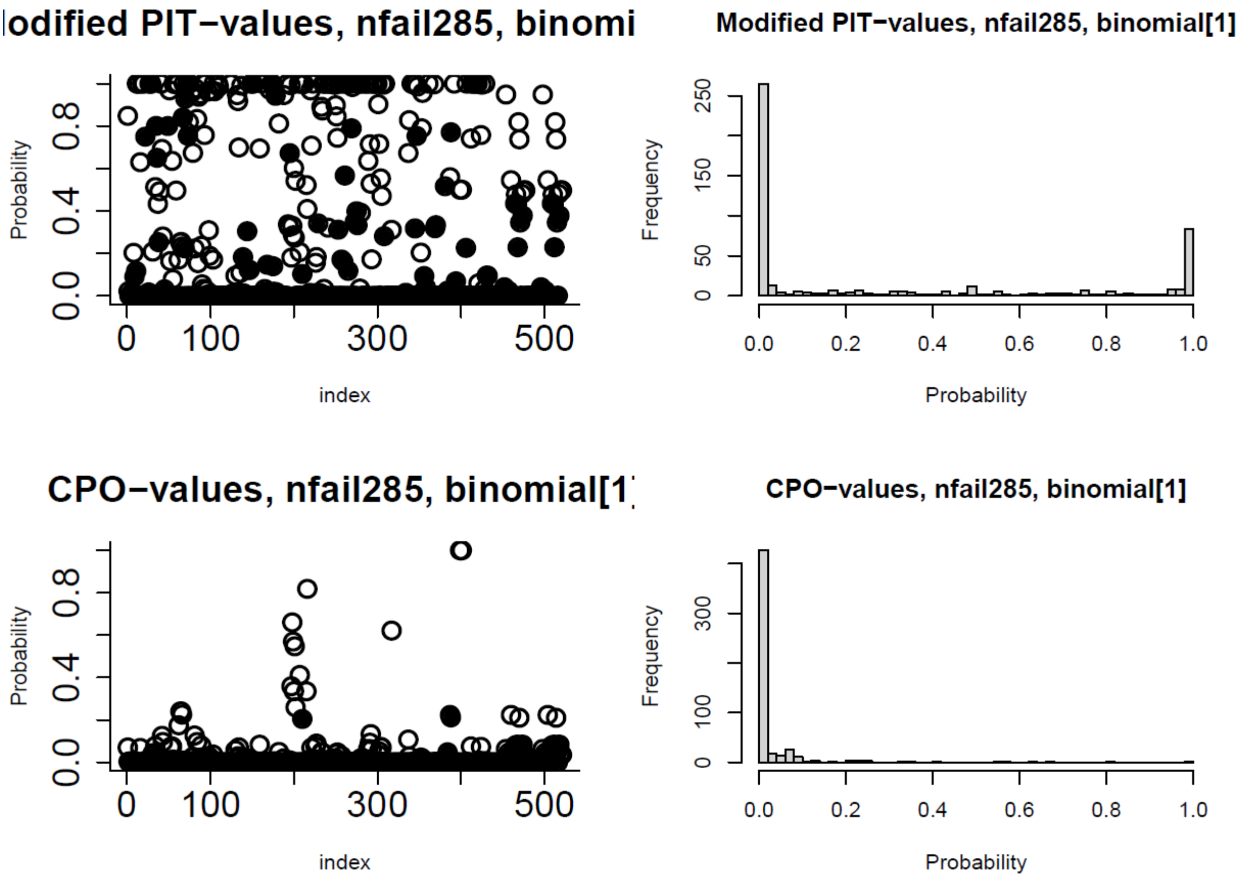

Strongyloides

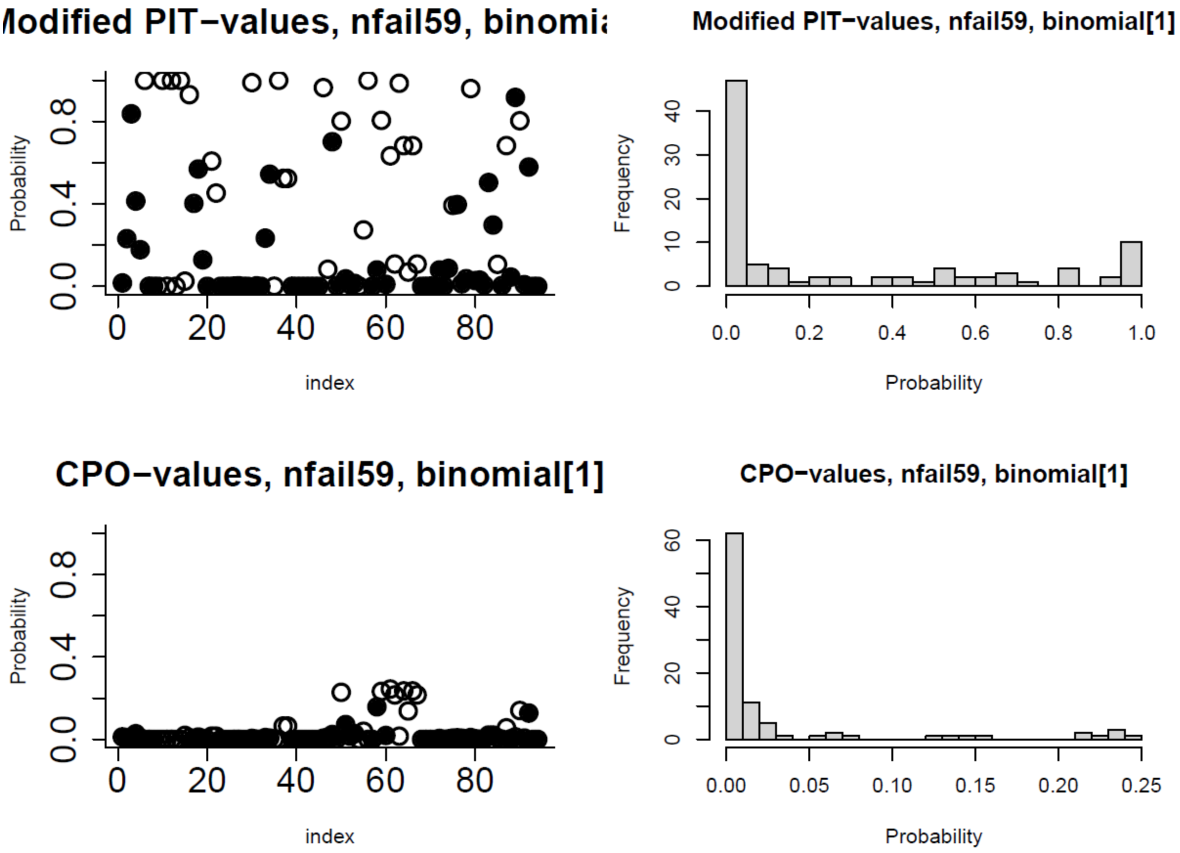

Hookworm

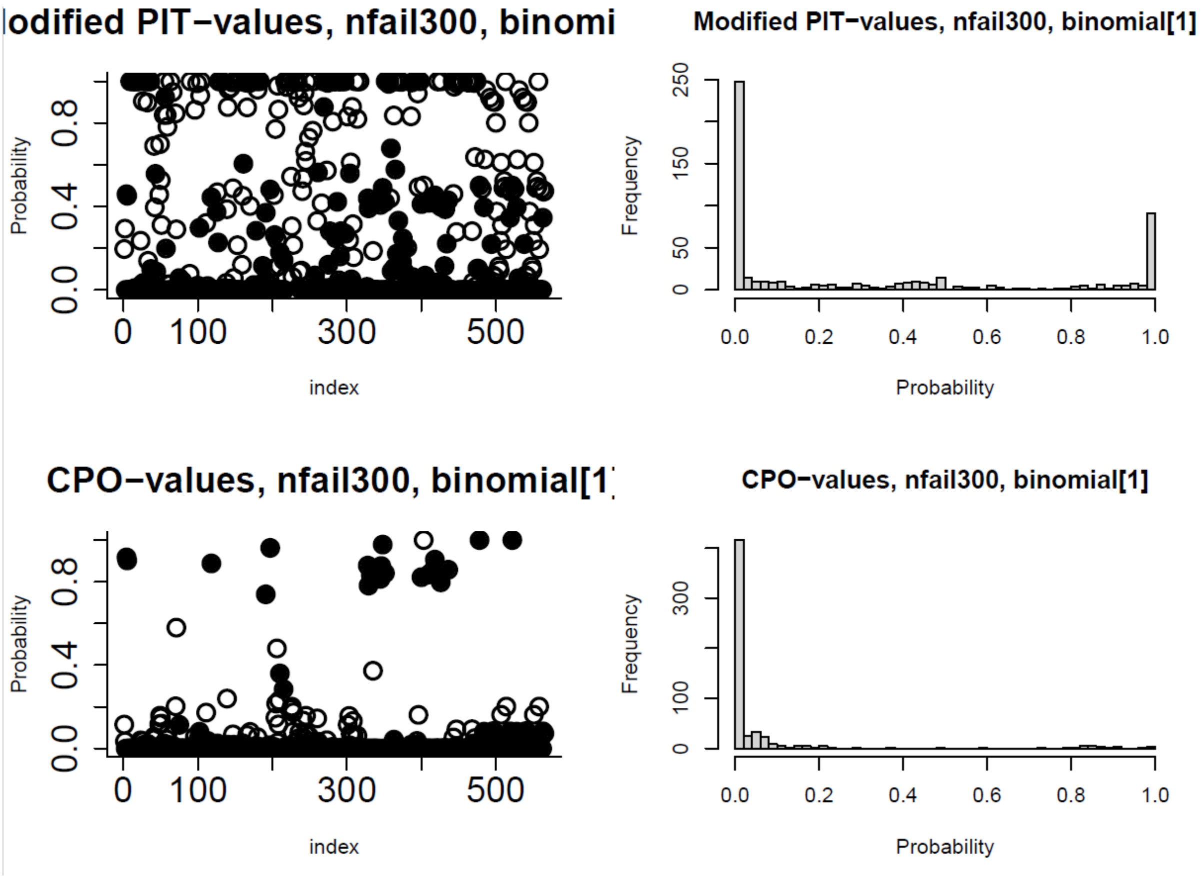
odified PIT−values, nfail300, binomi
Probability
0.0 0.4 0.8
0 100 300 500
index
Modified PIT−values, nfail300, binomial[1]
Frequency
0 50 150 250
0.0 0.2 0.4 0.6 0.8 1.0
Probability
CPO−values, nfail300, binomial[1
Probability
0.0 0.4 0.8
0 100 300 500
index
CPO−values, nfail300, binomial[1]
Frequency
0 100 300
0.0 0.2 0.4 0.6 0.8 1.0
Probability

T. trichiura

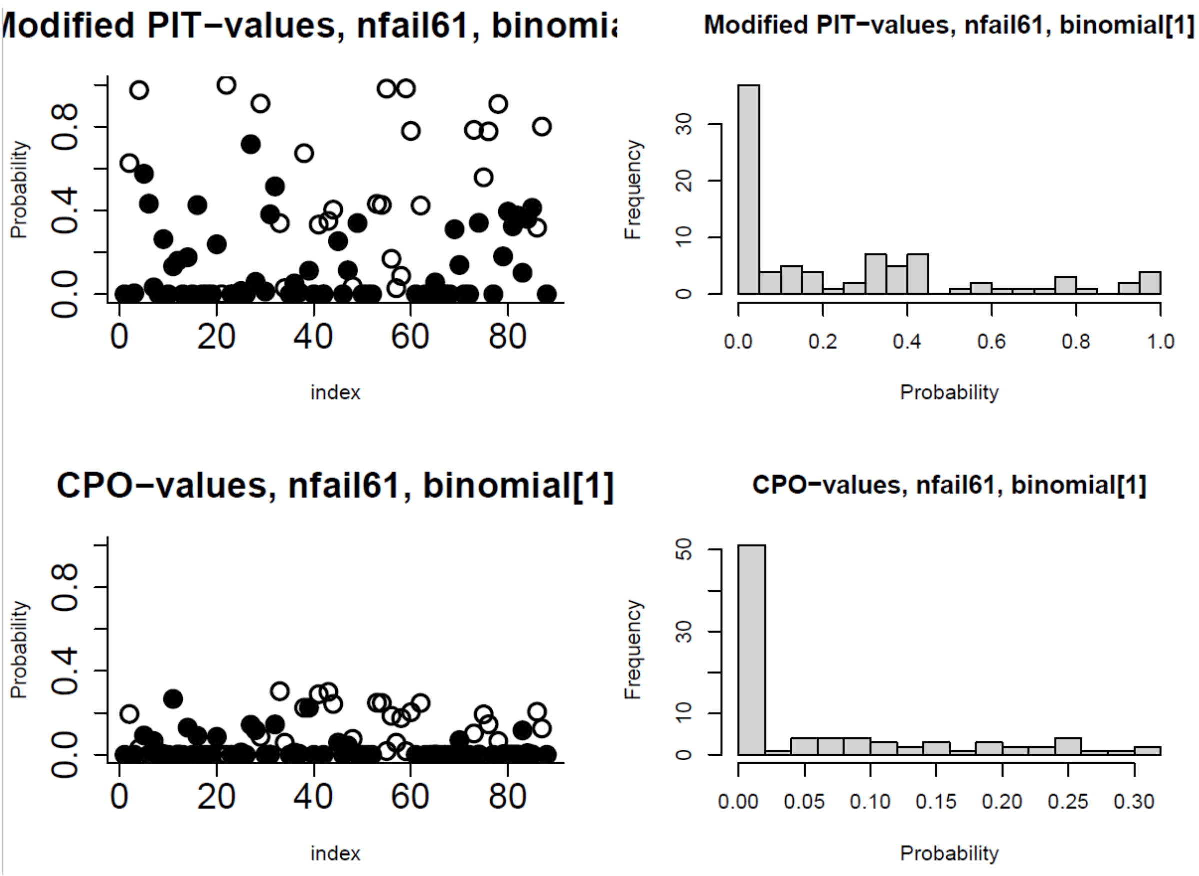

Modified PIT−values, nfail61, binomial[1]
Probability
index
Frequency
Probability
CPO−values, nfail61, binomial[1]
Probability
index
Frequency
Probability

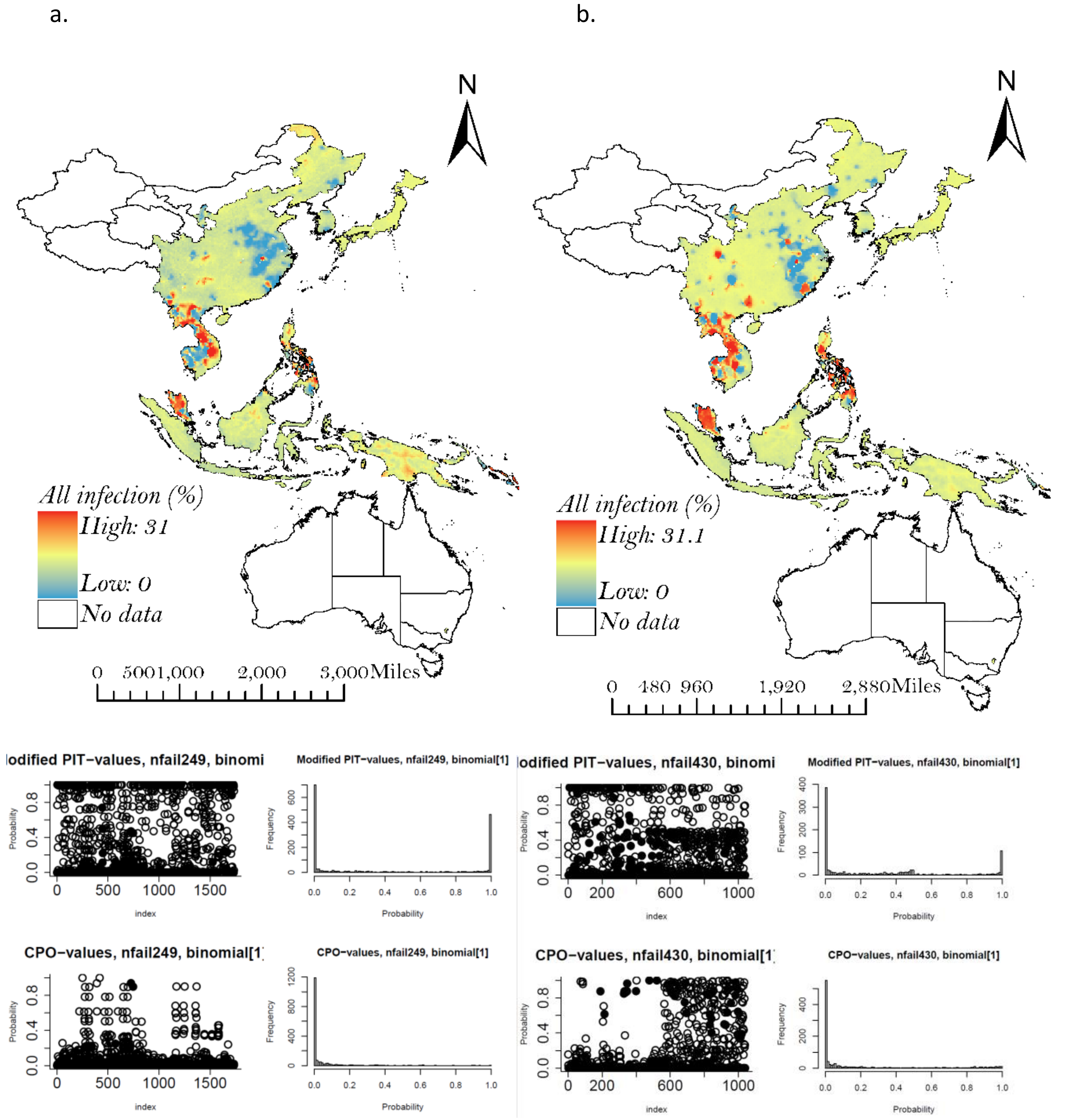

Supplementary Figure 6 : Predicted geographical distribution of STH infections and model validation plots

a) correlation across different landmass set zero b) correlation set as a direct function of straight line.

References

1. Page MJ, McKenzie JE, Bossuyt PM, Boutron I, Hoffmann TC, Mulrow CD, et al. The PRISMA 2020 statement: an updated guideline for reporting systematic reviews. International journal of surgery. 2021;88:105906.
2. Gilmour B, Wangdi K, Restrepo AC, Tsheten T, Kelly M, Clements A, et al. Protocol for spatial prediction of soil transmitted helminth prevalence in the Western Pacific region using a meta-analytical approach. Systematic Reviews. 2024;13(1):55.
3. World Health Organization. Countries n.d.D [Available from: https://www.who.int/countries.
4. World Health Organization. Global Burden of Disease Regions used for WHO-CHOICE Analyses n.d. [Available from: https://www.who.int/choice/demography/regions/en/.
5. Wells GA SB, O'Connell D, Peterson J, Welch V, Losos M, et al. The Newcastle-Ottawa Scale (NOS) for assessing the quality of nonrandomised studies in meta-analyses. 2014.
6. Savioli L, Daumiere D, Savioli D. Accelerating work to overcome the global impact of neglected tropical diseases: a roadmap for implementation. Geneva: World Health Organization. 2012:1-42.
7. WorldClim. Global climate and weather data. 2020.
8. Farr TG, Kobrick M. Shuttle Radar Topography Mission produces a wealth of data. Eos, Transactions American Geophysical Union. 2000;81(48):583-5.
9. Smith B, Sandwell D. Accuracy and resolution of shuttle radar topography mission data. Geophysical Research Letters. 2003;30(9).
10. Weiss DJ, Nelson A, Gibson H, Temperley W, Peedell S, Lieber A, et al. A global map of travel time to cities to assess inequalities in accessibility in 2015. Nature. 2018;553(7688):333-6.
11. Tatem AJ. WorldPop, open data for spatial demography. Scientific data. 2017;4(1):1-4.
12. SoilGrids — global gridded soil information. Available at: https://www.isric.org/explore/soilgrids. Access date. 02/02/2024 [Internet].
13. Worldwide land cover mapping. Available at: https://esa-worldcover.org/en. Access date: 02/02/2024 [Internet].
14. Areas GA. GADM database of global administrative areas. Global Administrative Areas. 2012.
15. Lewis PO, Xie W, Chen M-H, Fan Y, Kuo L. Posterior predictive Bayesian phylogenetic model selection. Systematic biology. 2014;63(3):309-21.
